# Enabling Distributed Intelligence with Ferroelectric Multifunctionalities

**DOI:** 10.1002/advs.202103842

**Published:** 2021-10-31

**Authors:** Kui Yao, Shuting Chen, Szu Cheng Lai, Yasmin Mohamed Yousry

**Affiliations:** ^1^ Institute of Materials Research and Engineering A*STAR (Agency for Science, Technology and Research) 2 Fusionopolis Way Innovis 138634 Singapore

**Keywords:** dielectric, ferroelectric, memory, piezoelectric, pyroelectric, self‐power, sensor

## Abstract

Distributed intelligence involving a large number of smart sensors and edge computing are highly demanded under the backdrop of increasing cyber‐physical interactive applications including internet of things. Here, the progresses on ferroelectric materials and their enabled devices promising energy autonomous sensors and smart systems are reviewed, starting with an analysis on the basic characteristics of ferroelectrics, including high dielectric permittivity, switchable spontaneous polarization, piezoelectric, pyroelectric, and bulk photovoltaic effects. As sensors, ferroelectrics can directly convert the stimuli to signals without requiring external power supply in principle. As energy transducers, ferroelectrics can harvest multiple forms of energy with high reliability and durability. As capacitors, ferroelectrics can directly store electrical charges with high power and ability of pulse‐mode signal generation. Nonvolatile memories derived from ferroelectrics are able to realize digital processors and systems with ultralow power consumption, sustainable operation with intermittent power supply, and neuromorphic computing. An emphasis is made on the utilization of the multiple extraordinary functionalities of ferroelectrics to enable material‐critical device innovations. The ferroelectric characteristics and synergistic functionality combinations are invaluable for realizing distributed sensors and smart systems with energy autonomy.

## Graphical abstract

Ferroelectric materials possess extraordinary multiple signal or energy conversion and storage functions, including high dielectric permittivity, switchable spontaneous polarization, piezoelectric, pyroelectric, and bulk photovoltaic effects. The characteristics of ferroelectrics, and the synergized combinations of their multiple functionalities are invaluable for realizing distributed wireless sensors and smart systems with energy autonomy.

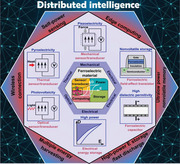

## 1 Introduction

The modern concept of basic ferroelectricity started with the discovery of the peculiar dielectric properties in Rochelle salt about 100 years ago.^[^ ^1^ ^]^ With highly responsive polar structure and spontaneous polarization electrically switchable, many functional properties have been found in ferroelectric materials, such as high dielectric permittivity, pyroelectric, piezoelectric, and bulk photovoltaic properties.^[^ ^2^, ^3^ ^]^ While these properties also exist in other non‐ferroelectric materials with asymmetric or polar structure, even before the discovery of ferroelectricity, the ferroelectric materials often exhibit extraordinarily stronger responses to the external stimuli, largely attributed to their relatively smaller energy barriers among their multiple thermodynamically stable polarization states. Therefore, ferroelectric materials have achieved many technically important applications due to the competitiveness of these performance properties.^[^ ^4^ ^]^ The well‐established commercial applications with billions of US$ industry today include dielectric capacitors with high permittivity, electromechanical sensors and ultrasound transducers with piezoelectric effect, thermal detectors and imaging sensors with pyroelectric effect, ferroelectric memories with their switchable spontaneous polarization.^[^ ^5^, ^6^ ^]^

With the rapid spread of cyber‐physical interactive digital technology and advancement in IoT (Internet of Things), a vision of the future world with connectivity among a trillion devices is formed with consensus.^[^ ^7^, ^8^, ^9^, ^10^, ^11^, ^12^ ^]^ Before such an IoT vision comes true, implementation of tremendous number of smart sensors is required, and edge computing is desired for realizing effective distributed intelligence. While the distributed sensors and decentralized computing are pervasive, the problem of effectively connecting and powering the sensors and edge computing are becoming one of the major challenges.^[^ ^13^, ^14^, ^15^, ^16^, ^17^ ^]^ The sensors and smart systems with following features are demanded for achieving the distributed intelligence as desired for the coming digital cyber‐physical connections and IoT applications:

(1) Low or even zero power consumption, or energy autonomy enabled by power harvested from the ambient, because batteries or socket power may not be available or not realistic to the sensors widely distributed;
(2) Wireless communication, as wire connection to each of the distributed sensors is becoming a nightmare for the users;
(3) Signal processing at the edge, even under intermittent power supply condition with uncertain availability, such as the energy harvested from the environment; and
(4) Robust and durable operation, as always desired for all intelligent devices.

**Figure 1:**
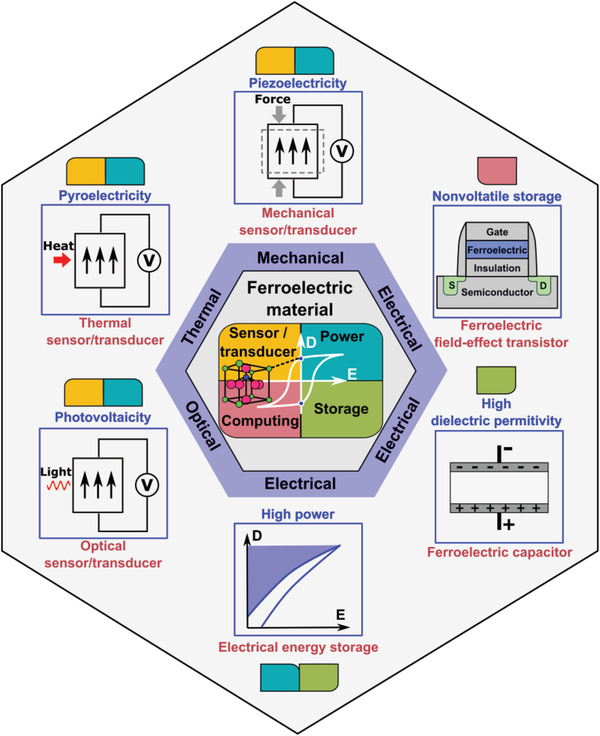
Multiple functionalties of ferroelectric materials as sensors, energy harvesting transducers, energy storage capacitors, and nonvolatile memories, with the potential for realizing distributed intelligence with energy autonomy.

We believe ferroelectric materials exhibit the great potentials to enable the sensors and smart systems with the device features as demanded above for the future cyber‐physical interaction technologies and IoT applications. Here the recent progresses on ferroelectric materials and their enabled device functions with promising contributions to realizing energy autonomous sensors and smart systems towards distributed intelligence are reviewed, starting with an analysis on the basic characteristics of ferroelectrics. An emphasis has been made on the utilization of the multiple extraordinary functionalities of ferroelectrics, as summarized in **Figure** **1**, including multiple self‐powered sensing and energy harvesting mechanisms, low power nonvolatile memory, energy storage, and their synergistic combinations to enable energy autonomous smart systems.

## 2 Ferroelectrics for Sensors

### 2.1 In Principle Self‐Powered Sensing Mechanisms with Ferroelectric Materials

In most smart systems and IoT applications, signal processing is performed through electric circuitry, and electrical signal is the most preferred form of output from a sensor, which can be directly recognized and processed. With the polar structure and responsive electrical polarization, ferroelectric materials possess an inherent characteristic that can convert the external stimuli in various forms of energy into electricity, such as converting mechanical energy into electricity through piezoelectric effect,^[^ ^18^, ^19^ ^]^ thermal energy into electricity through pyroelectric effect,^[^ ^20^ ^]^ optical energy into electricity through bulk photovoltaic effect.^[^ ^21^ ^]^ Piezoelectric effect has been widely used to produce electromechanical sensors, including pressure sensors, shock sensors, accelerometers, acoustic sensors, microphone, and hydrophone.^[^ ^22^ ^]^ Pyroelectric effect has been used in temperature meters, infrared detectors and imaging sensors.^[^ ^23^ ^]^ Bulk photovoltaic effect has been used to produce UV detector and dosimeter.^[^ ^24^ ^]^

It should be emphasized here that the ferroelectric sensors as listed above are characterized by the ability to generate electrical output signals via direct conversion of mechanical, thermal, and optical energy, respectively, without requiring any other external energy input in principle. This is different from many other sensors. An accelerometer for vibration measurement can be used as an example to explain this point. Besides piezoelectric effect, accelerometers are also produced from capacitive or resistive effects, in which the sensing principle is based on the changes of capacitance^[^ ^25^ ^]^ or resistance^[^ ^26^ ^]^ in response to the vibration. In these two cases, an electric bias is usually required to determine the capacitance and resistance changes, which means an external energy source is necessary according to the basic operation principle. In contrast, no external electrical bias is required for generating the electrical output for a piezoelectric accelerometer, although an electrical supply is often implemented for amplifying and processing the signals, so that the existing ferroelectric sensors are usually not self‐powered in most practical applications.^[^ ^27^ ^]^

### 2.2 A Large Variety of Parameters Detectable by Ferroelectric Sensing

The presence of a damage or defect, such as crack or delamination, could be measured to indicate the integrity of a mechanical structure; many physical and chemical parameters, such as temperature, pressure, vibration, light, and gas emission, are measured to monitor the condition of a machine, infrastructure, or environment. Ferroelectric materials have shown the ability and value in determining almost all these abnormities and parameters. With its competitive piezoelectric effect, ferroelectric materials are widely used for producing accelerometers, microphones, hydrophones, acoustic transducers, pressure and shock sensors.^[^ ^28^, ^29^ ^]^ Most of the passive acoustic emission sensors and active ultrasonic transducers are made of ferroelectric materials, and they are widely used for monitoring structural integrity by detecting or measuring crack, delamination, plastic deformation, distance and dimension.^[^ ^30^, ^31^, ^32^, ^33^, ^34^, ^35^, ^36^ ^]^ Ferroelectric materials are widely applied to produce electromechanical resonators, surface acoustic wave (SAW), bulk acoustic wave (BAW) devices, etc., which are used to measure various physical parameters including temperature, pressure, and vibration,^[^ ^37^, ^38^, ^39^ ^]^ and for detecting chemicals and biological species as well.^[^ ^40^, ^41^ ^]^ Temperature monitoring, infrared detection and imaging are realized with wide applications through pyroelectric effects of ferroelectric materials.^[^ ^42^ ^]^ Robust UV sensors including dosimeters are realized from ferroelectric films by utilizing their bulk photovoltaic effect for monitoring UV irradiation with stable operation under high intensity.^[^ ^43^ ^]^ Magnetic sensors are produced by utilizing multiferroic materials or multiferroic composite structure.^[^ ^44^, ^45^ ^]^ Therefore, ferroelectric sensors can be used for detecting and measuring a large variety of parameters, and are very valuable for various structural and condition monitoring with established track record in industry.

Since self‐powered sensing functionalities are concerned in this review, **Table** **1** provides the performance properties of typical ferroelectrics for sensing multiple basic physical parameters, with emphasis on sensitivity in term of electric output before external circuit amplification, including piezoelectric acceleration, ultrasound/pressure, optical and pyroelectric sensing. A key challenge in realizing self‐powered sensing system is how to drive the entire operation system with the small primary electrical sensor output, or to utilize the energy harvested from separate energy harvesters, as to be discussed in the next section.

**Table 1:** Performance properties of ferroelectric materials in term of electric output for piezoelectric acceleration, ultrasound/pressure, optical and pyroelectric sensing

Sensing Type	Sensing Materials/Devices	Sensitivites*	Detectable/Detectivity/Error	Reference Remarks
Piezoelectric Acceleration	BTO nanowires	50 mV g^−1^ (300 Hz)	≈0.005 g	Not in resonance^[^ ^46^ ^]^
Sensing	PZT thin film	8.9 ‐17 mV g^−1^ (20–600 Hz)	Error: < 10%	Cantilever structure^[^ ^47^, ^48^ ^]^
	PVDF film	60.5 mV g^−1^ (13 Hz)	0.01 g, 0.1 Hz	Cantilever structure^[^ ^49^ ^]^
	PVDF film accelerometer	50 mV g^−1^ (90 Hz)		Commercial Product^[^ ^50^ ^]^
Piezoelectric Ultrasonic/	PZT thin film	0.48 mV kPa^−1^ (8 MHz)		Diaphram (60 µm in diameter); water^[^ ^51^ ^]^
Pressure Sensing	PZT ceramic	4.28 mV kPa^−1^ (2.34 MHz)		Commerical probe; water^[^ ^52^ ^]^
	PZT thin film‐MOSFET	≈0.8 µV Pa^−1^ (10 to kHz)	0.005 Pa	Flexible 400 nm‐PZT on skin^[^ ^53^ ^]^
	P(VDF‐TrFE) fiber sheet	∼800 µV Pa^−1^ (≈1 Hz)	0.1 Pa	Electrospun fibers^[^ ^28^ ^]^
	BTO‐PVDF/GO core‐shell	10 mV kPa^−1^ (2 Hz)	Error: ∼±5%	Coaxial electrospun fibers^[^ ^54^ ^]^
Photovoltaic	BTO crystal	10 µA W^−1^	≈1 × 10^8^ Jones	Time: 200 ps^[^ ^55^ ^]^
Optical Sensing	BTO thin film	0.2 µA W^−1^	≈4–6 × 10^5^ Jones	Time: 0.6 s^[^ ^56^ ^]^
	BTO thin film	150 µA W^−1^		Time: 10s ns^[^ ^57^ ^]^
	NiO/PLZT heterojunction	180 µA W^−1^	3.69 × 10^9^ Jones	Time: 0.3 s^[^ ^58^ ^]^
	PZT with Schottky barrier effect	94 mA W^−1^	Not available	Ultra‐thin (12 nm)^[^ ^59^ ^]^
Pyroelectric	PZT thin film	2.1 V W^−1^	1.49 × 10^8^ Jones	Epitaxial film^[^ ^60^ ^]^
Sensing	PZT thin film	1.7 V W^−1^		Multilayer^[^ ^61^ ^]^
	LNO film	193 V W^−1^		Single‐crystal^[^ ^62^ ^]^

* ^Before amplification effect by external electronic.^

Jones: cm Hz^1/2^ W^−1^, BTO: BaTiO_3_, PZT: Pb(Zr,Ti)O_3_, LNO: LiNbO_3_, GO: graphene oxide.

## 3 Ferroelectrics for Energy Harvesting

**Figure 2:**
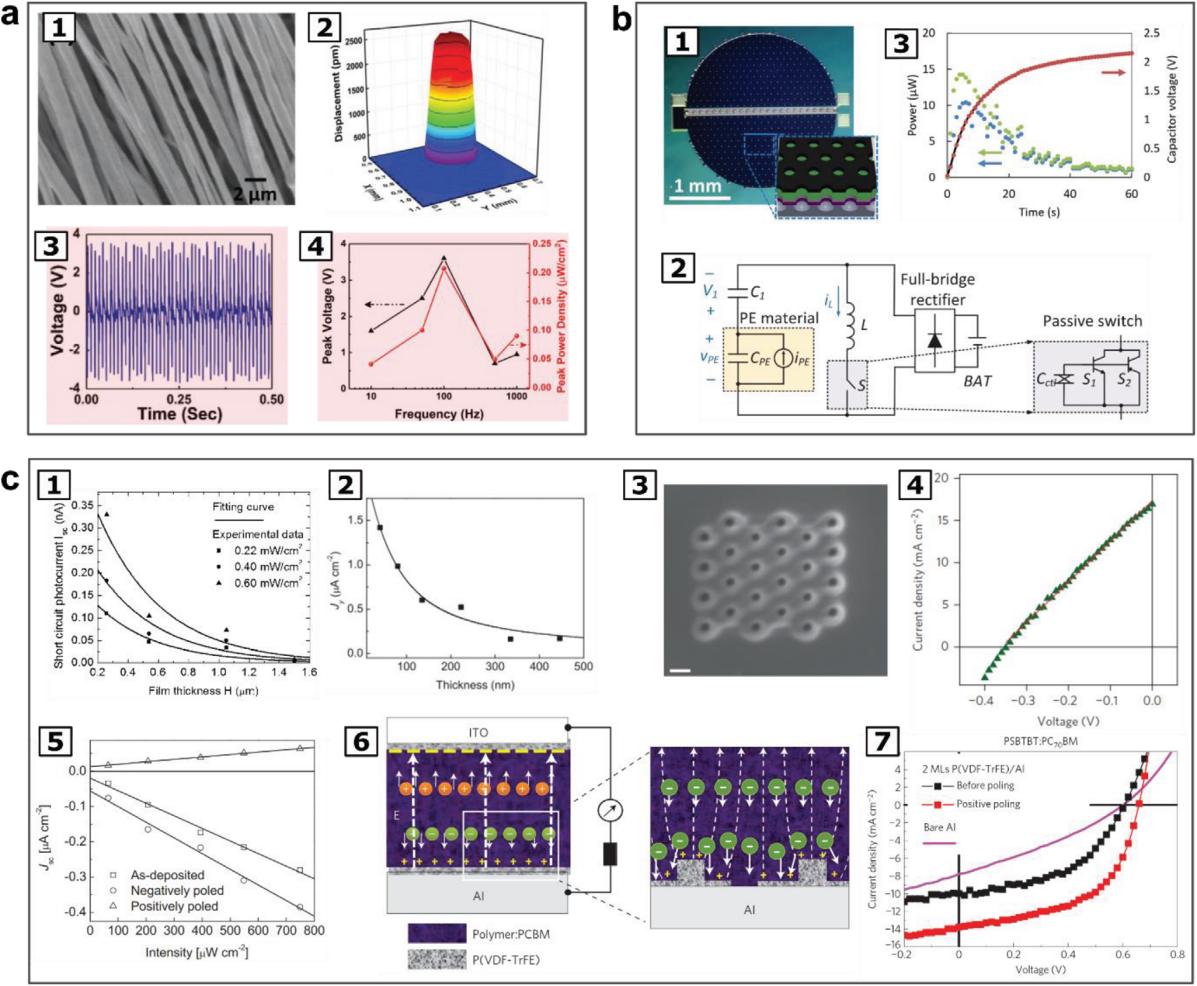
Ferroelectric materials and mechanisms for energy conversions. a) Piezoelectric effect of electrospun PVDF fibers for kinetic energy harvesting: a1) SEM image of the electrospun PVDF fibers. a2) The electric field excited vibration of a PVDF fiber film characterized by a laser scanning vibrometer. a3) Voltage versus time graph measured for the PVDF fiber film under excitation condition of 100 Hz and 3.0 g. a4) Peak voltage and peak power density of the PVDF fiber film at different frequencies. Reproduced with permission.^[^ ^76^ ^]^ Copyright 2020, Wiley‐VCH. b) Thermal energy harvesting from pyroelectric effect: b1) Photograph of a pyroelectric energy harvester (inset: the device's cross‐sectional structure). b2) Circuit for power harvesting in the pyroelectric charging system. b3) Measurement result of the pyroelectric energy harvesting system to charge a capacitor (100 µF): Red: voltage on the capacitor, Blue: average power at the storage capacitor, Green: average power in consideration of power loss on the rectifier. Reproduced with permission.^[^ ^84^ ^]^ Copyright 2018, Elsevier. c) Photovoltaic effect in ferroelectrics: c1) Short circuit photocurrent in ferroelectric PbLaZrTiO_3_ (PLZT) thin films at different thicknesses. Reproduced with permission.^[^ ^88^ ^]^ Copyright 2007, AIP Publishing. c2) Bulk photovoltaic current density in ferroelectric BiFeO_3_ thin films at different thicknesses. Reproduced with permission.^[^ ^91^ ^]^ Copyright 2011, American Physical Society. c3) Photo of an array of 24 focused ion beam‐milled holes (10 nm deep) through a SiO_2_ layer (50 nm thick) into a poled and single domain BaTiO_3_ (001) bulk crystal forming a photovoltaic device. Scale bar: 200 nm. c4) The effective current density–voltage response of the photovoltaic device under AM1.5 G illumination (intensity: 100 mW cm^−2^). Reproduced with permission.^[^ ^94^ ^]^ Copyright 2016, Springer Nature. c5) Photovoltaic response of ITO/BiFeO_3_(170 nm)/SrRuO_3_/SrTiO_3_(001): change of short‐circuit current density (J_sc_) with light intensity. Reproduced with permission.^[^ ^95^ ^]^ Copyright 2010, Wiley VCH. c6) Working principle of ferroelectric organic photovoltaic device: the structure of polymer photovoltaic device phenyl‐C61‐butyric acid methyl ester (PCBM) with ferroelectric interfacial layers (P(VDF/TrFE)) showing the electric field induced by ferroelectric layer with the electric field‐assisted charge extraction. The diagram at right shows the electric‐field distribution and movement of electrons through the P(VDF‐TrFE) near the aluminium electrode. c7) Improvement of photocurrent response by the ferroelectric layer in a PSBTBT:PC70BM device. Reproduced with permission.^[^ ^99^ ^]^ Copyright 2011, Springer Nature. c1–c4) demonstrate the improved photovoltaic efficiency in low dimensional ferroelectrics and ferroelectric‐involved interfaces; c5) An example of photovoltaic response under visible light for ferroelectric materials with smaller energy bandgap; c6,c7) give an example of enhancing charge separation and transfer, and thus increasing the efficiency of photovoltaics by taking use of the depolarization field of a ferroelectric.

The functions of directly converting multiple forms of energy into electricity as seen in many ferroelectric sensors are utilized for harvesting various energy from the environment, as illustrated with examples in **Figure** **2**. The energy conversion efficiency and the obtained electrical energy or power output are critical to low power or energy autonomous system applications. The advantages, constraints, and recent progresses for harvesting different forms of energy with ferroelectric materials are analyzed below.

### 3.1 Kinetic Energy Harvesting by Piezoelectric Effect

There are different ways to convert kinetic energy into electricity, including electrostatic, electromagnetic, and piezoelectric approaches. Among them, piezoelectric technologies using the material without a center of structural symmetry have several advantages, including simplified electromechanical structure, easy to be miniaturized, high energy density, particularly at small sizes, and without requiring separate voltage source.^[^ ^63^, ^64^ ^]^ One technical constraint in a practical application is that the mechanical input energy must be transformed into the strain of the piezoelectric material for generating the electricity. It is thus essential to well mechanically couple the piezoelectric material with the mechanical energy source, such as a vibrating plate, a rotating shaft or a footstep, for effectively inducing the piezoelectric material strain. The square of electromechanical coupling coefficient (*k^2^*) is used for evaluating how effectively mechanical energy can be converted to electricity, which is determined by the material and the mode of the strain. For realizing highly efficient kinetic energy harvesting through piezoelectric effect, piezoelectric materials with high electromechanical coupling coefficient are demanded in general. The unconverted mechanical energy largely retains as mechanical energy, and the loss of the energy is small during the electromechanical conversion process, typically below a few percent mainly due to dielectric loss, mechanical loss, and other electromechanical coupling loss.^[^ ^65^ ^]^ In some cases, a figure of merit (FOM) of d^2^/ε_r_ for describing kinetic energy harvesting performance is used, where *ε_r_* is the relative dielectric permittivity, and *d* is the piezoelectric strain or charge coefficient.^[^ ^66^ ^]^ Since ferroelectric materials have significantly higher piezoelectric coefficient and electromechanical coupling coefficient than non‐ferroelectric piezoelectric materials, they are the main stream of material choices for piezoelectric energy harvesting. In addition, many structures are made with dedicated design to improving the kinetic energy harvesting performance. For examples, nonlinear mechanical structure was designed for harvesting wideband vibrations.^[^ ^67^ ^]^ To enhance mechanical coupling effect through better mechanical impedance match at low frequency range, reducing mechanical impedance by the use of porous and soft ferroelectric materials was implemented.^[^ ^68^, ^69^, ^70^ ^]^ Multilayer structure was used to significantly improve the electrical output, not only by simply increasing the area and volume of the active piezoelectric layer, but also by improving the electric impedance match between the piezoelectric transducer and load circuit.^[^ ^71^, ^72^ ^]^

Triboelectric effect, such as involving electrets and material surfaces with different electron affinity is also utilized for producing electricity from mechanical movement. While the triboelectric effect can produce larger electrical output, particularly at low frequency,^[^ ^73^, ^74^ ^]^ the durability under operation cycling and stability in certain atmosphere such as with high humidity may be an issue in some practical applications. In contrast, the piezoelectric effect originates from the ferroelectric behaviors corresponding to thermodynamically stable state and has the established track record of excellent durability and stability. By designing the material or structure with constructive piezoelectric and triboelectric effects, kinetic energy harvesting capabilities with enhanced effectiveness over broad frequency range could be realized.^[^ ^75^, ^76^ ^]^ An example is given in Figure 2a, where kinetic energy harvesting was enhanced by piezoelectric and triboelectric effects constructively in electrospun PVDF fibers. A review focusing on piezoelectric energy harvesting can be referred to the recent reference.^[^ ^77^ ^]^

### 3.2 Thermal Energy Harvesting with Pyroelectric and Temperature Dependent Dielectric Effects

With large temperature dependence of the spontaneous polarization, ferroelectric materials can show a high pyroelectric coefficient. There are different FOMs proposed for evaluating the pyroelectric energy harvesting performance, and almost all of them are proportional to p^2^/ε, where *p* is pyroelectric coefficient and *ε* is dielectric permittivity.^[^ ^78^ ^]^ When 0.5Z_pryo_T_h_η_c_<<1, with *Z_pyro_* representing a temperature dependent factor determined by the pyroelectric coefficient, dielectric permittivity and specific heat, the energy conversion efficiency for the cycle in an energy converter can be estimated by Equation (1) below

(1) $$\eta_{pyro}\approx \frac{\gamma^{2}}{C_{V}\varepsilon }T_{h}\eta_{C}$$

where *γ* is the pyroelectric coefficient, *η_C_* the Carnot cycle efficiency, *C_V_* the specific heat of the unit volume, and *T_h_* temperature of the heat source.^[^ ^79^ ^]^ The conversion efficiency for high performance pyroelectric materials could reach a few percent of the Carnot cycle efficiency. With solar energy conversion to the electricity as an example, assuming the amplitude of temperature change at 10 K, the pyroelectric conversion efficiency only reaches ≈0.3%. With solar heat flux density of 10 W cm^−2^ order under direct sunshine, the electric power density could be around 0.3 mW cm^−3^, orders of magnitude lower compared to solar cell. However, study showed that the efficiency of pyroelectric effect could be significantly enhanced under certain conditions. Morozovska et al reported their analyses that pyroelectric coefficient increased with radius decrease of ferroelectric wires, in which a giant pyroelectric current is resulted from a small size‐driven pyroelectric coupling improvement, and the efficiency of the pyroelectric energy harvesting at low temperature could potentially approach the Carnot limit.^[^ ^80^ ^]^

Pyroelectric energy harvester has not been widely used for energy harvesting because its relatively lower efficiency compared to solar cell or thermoelectric effect,^[^ ^81^, ^82^ ^]^ as most ambient temperature fluctuations are slow without high frequency thermal cycling. However, its output is still adequate for some low power electronic devices and microsystems. For example, motivated by the attainment of sustainable power for wireless sensor networks, a complete pyroelectric energy management system with functions of wireless thermal energy harvesting (with maximum harvested power of 13.1 µW) and storage was demonstrated, as introduced in Figure 2b. The system included a laser as the heat source, a Pb(Zr,Ti)O_3_ (PZT) thin film pyroelectric device, a power interface for optimizing pyroelectric conversion cycle, and a battery or a capacitor as the energy storage component.^[^ ^83^, ^84^ ^]^ The results showed the potential of pyroelectric energy harvesting from waste‐heat for supplying power to low power sensors. The use of the laser without physical contact in the example indicated the potential of wireless power transmission operation. As the pyroelectric effect coexists with many other energy harvesting functions, such as piezoelectric and bulk photovoltaics effects, appropriate design could enhance energy harvesting performance through constructive effects as described in Section 5.^[^ ^85^ ^]^

It should be noted that since many ferroelectrics have a strong temperature dependence of the large dielectric permittivity, particularly around phase transition temperatures, they could generate electricity due to the electrical charging and discharging with temperature change even without pyroelectric properties. Such mechanism also works effectively in a paraelectric phase for ferroelectrics or antiferroelectrics with significantly temperature dependent dielectric permittivity near a phase transition temperature. The efficiency could reach a few percent for a properly selected material in selected operation temperature range.^[^ ^79^ ^]^

### 3.3 Optical Energy Harvesting with Photovoltaic and Photochemical Effects

Bulk photovoltaic effect (BPVE) in a conventional bulk ferroelectric material is attributed to asymmetric momentum distribution of the photo‐induced nonthermalized electrical charges in non‐centrosymmetric structure. While a large photovoltage can be generated with switchable polarity, the efficiency of BPVE in bulk ferroelectrics is very low for energy harvesting application, typically 10^−6^ to 10^−4^, due to the much shorter lifetime of electrons in the nonthermalized state in comparison with nonequlibrium thermalized electrons.^[^ ^21^, ^86^, ^87^ ^]^ Thus, there is no much attention paid on BPVE in conventional bulk materials for light energy harvesting. The interests in exploring photovoltaics involving ferroelectrics for energy harvesting mainly started in the recent 15 years, inspired by discoveries and progresses in the following three aspects. Firstly, photovoltaic efficiency in low dimensional ferroelectrics and ferroelectric‐involved interfaces was significantly improved over the conventional bulk ferroelectric materials by orders of magnitude.^[^ ^88^, ^89^, ^90^, ^91^, ^92^, ^93^, ^94^ ^]^ Secondly, some ferroelectric materials with small energy bandgap exhibited photovoltaic response under visible light,^[^ ^95^ ^]^ and a high efficiency up to 6% in response to red light was reported in ABO_3_ perovskite structure with B‐site cationic ordering.^[^ ^96^, ^97^, ^98^ ^]^ Thirdly, by making use of the depolarization field in ferroelectrics to promote charge separation and transfer, the efficiency of photovoltaics in other semiconductor materials or their interfaces with the ferroelectric material was substantially improved in the composite structures comprising the semiconductor and ferroelectric materials.^[^ ^99^, ^100^, ^101^, ^102^ ^]^ Figure 2c provides several examples about the ferroelectric photovoltaic effect at low dimensions, under visible light, or in the composite with improved charge separation and efficiency by utilizing the depolarization field in a ferroelectric material.

These progresses have raised interests to look into the potential of ferroelectric materials of low dimensions, narrowed banded, and hybrid structures for harvesting optical energy, rather than the conventional bulk ferroelectric materials. It should be noted that a photovoltage much larger than interfacial energy barrier in typical semiconductors could still be obtained in thin films, such as 7.0 V demonstrated with in‐plane polarized ferroelectric thin film.^[^ ^103^ ^]^ A. Perez‐Tomas et al showed ferroelectric PZT/*β*‐Ga_2_O_3_ structure exhibited BPVE with switchable voltages above‐bandgap under white light illumination with a large photovoltaic field up to 0.7 MV cm^−1^.^[^ ^104^ ^]^ In addition, as ferroelectric materials in contact with semiconductors or materials with varied valence states could promote the migration of photogenerated carriers or redox reaction by appropriately tuning energy band structure at the interfaces, they showed the ability in improving the solar photochemical efficiency.^[^ ^105^, ^106^, ^107^ ^]^

In general, use of ferroelectric materials for harvesting optical energy is not as popular as semiconductor materials, mainly due to the fundamental low efficiency of BPVE and high impedance of ferroelectrics limiting the photocurrent. However, by the combination of BPVE and other ferroelectric properties, not only energy output can be enhanced by harvesting various forms of energy from the environment, but also battery‐less and wireless optical sensors as described in Section 5.

**Table** **2** provides the energy outputs from multiple energy harvesting mechanisms of ferroelectric materials, as reported in the recent years, including converting kinetic energy, thermal energy, and optical energy into electricity through piezoelectric, pyroelectric, and photovoltaic effect, respectively. Since there are no standards established for the investigations of various ferroelectric energy harvesting mechanisms and performance, the testing conditions for the reported results are very different. It is very challenging to make direct comparisons for the performance of all the different materials. Nevertheless, several features from the results in Table 2 should be highlighted. Firstly, the values of output power density, determined either in term of per unit area or volume, vary over a large range up to several orders of magnitude. For a specific application, not only the material properties, but also the loading condition and auxiliary architecture significantly affect the output power performance. For kinetic energy harvesting through piezoelectric effect with the large variety of mechanical loadings, the designs of the corresponding mechanical structure, such as appropriate implementation of proof mass, cantilever, cymbal or composite structure, for achieving electromechanical impedance match or effective mechanical coupling is critical to generating large power output. For thermal energy harvesting through pyroelectric effect, additional electric field is required for realizing Ericsson cycle and high power density above 1 mWcm^−3^. For many self‐powered sensor applications, such an external electric field may not be available and thus the power output is limited. Secondly, ferroelectric materials with low dimensional structural features could exhibit significantly improved power density. Compared with bulk materials, ferroelectric thin films produce power density with improved order of magnitude, particularly for thermal and optical energy harvesting by pyroelectric and photovoltaic effects, as summarized in Table 2.

**Table 2:** Outputs from multiple energy harvesting mechanisms in ferroelectric materials, including converting kinetic energy, thermal energy, and optical energy into electricity through piezoelectric, pyroelectric, and photovoltaic effect, respectively

Energy Sources /Mechanism	Material	Load/Input	Power Density		Reference RemarksV: output voltage; *I*: current density; *η*: efficiency
			Area (µW cm^−2^)	Volume (mW cm^−3^)	
Kinetic Energy / Piezoelectric	PZnT‐NT ceramic	Vibration (0.4 m s^−2^, 30 Hz)		0.13	Cantilever structure (t = 1 mm), V: 5.27 V ^[^ ^108^ ^]^
	KNFN ceramic	Vibration (1.2‐mm amplitude, 60 Hz)		0.38	Fe_2_O_3_‐KNN plate (4 × 6 × 0.4 mm^3^); V: 0.38 V ^[^ ^109^ ^]^
	PZT ceramic	Vibration (1 g, 514 Hz)	86.4	28.8	Thin plate (t = 30 µm); V: 2.7 V, *I*: 32 µA cm^−2 [^ ^110^ ^]^
	PMNT single crystal	Vibration (102 Hz, proof mass: 4.2 g)		29.6	Cymbal structure (t = 1 mm); V: 38 V ^[^ ^111^ ^]^
	BTO thin film	Tensile stress of 0.34 GPa		7	By sputtering (t = 300 nm); V: 1 V, *I*: 0.19 µA cm^−2 [^ ^112^ ^]^
	PZT thin film	Bending strain of ∼0.38%	17 400		By sol‐gel (t = 30 µm); V: 200 V, *I*: 150 µA cm^−2 [^ ^113^ ^]^
	KNN nanorod single crystal	Compressive force (9.8 N, 1 Hz)		0.10	By hydrothermal (L = 280‐550 nm); V: 0.38 V, *I*: 19 nA cm^−2 [^ ^114^ ^]^
	PZT nanowire	Compressive force		2.8	Epitaxial growth on Nb‐SrTiO_3_ (D = 500 nm, L = 5 µm); V: 0.7 V, *I*: 4 µA cm^−2 [^ ^115^ ^]^
	PZT nanofiber	Periodic pressure (0.53 MPa)	4911		Electrospun; V: 209 V, *I*: 23.5 µA cm^−2 [^ ^116^ ^]^
	PVDF nanofiber	Vibration (3 g, 100 Hz)	0.210		Electrospun (t = 150 µm); V: 3.6 V, *I*: 58 µA cm^−2 [^ ^76^ ^]^
	PVDF porous film	Sonic input (100 dB, 100 Hz)	1.56	0.17	By template (t = 5 µm); V: 2.6 V, *I*: 0.6 µA cm^−2 [^ ^68^ ^]^
	PVDF/BTO Composite	Vibration (10 g, 13 Hz)		0.027	Hot pressed (t∼0.3 mm); V: 37.5 V, *I*: 0.212 µA cm^−2 [^ ^117^ ^]^
	P(VDF‐HFP)/BTO composite	Compressive pressure (0.23 MPa)		480	Spin coated (t = 50 µm); V: 110 V, *I*: 10 µA cm^−2 [^ ^118^ ^]^
Thermal Energy/ Pyroelectric	PZT ceramic	Δ*T*: 300 to 308 K		0.004	Commercial (t = 1 mm); Q‐V cycle; load resistance: 1 GΩ ^[^ ^119^ ^]^
	PVDF film	Δ*T*: 300 to 323 K		0.300	Commercial (t = 11 µm); Q‐V cycle; load resistance: 50 GΩ ^[^ ^119^ ^]^
	PLZT ceramic	Δ*T*: 40 to 210°C, *ΔE*: 0 to 8.5 MV m^−1^, 0.06 Hz		48	(t = 200 µm); Ericsson cycle ^[^ ^120^ ^]^
	PNNZT ceramic	Δ*T*: 20 to 220°C, *ΔE*: 0.3 to 9.0 MV m^−1^, 0.09 Hz		78	Commercial (t = 0.2 mm); Ericsson cycle ^[^ ^121^ ^]^
	P(VDF‐TrFE) film	Δ*T*: 40 to 120°C, *ΔE*: 150 to 500 kVcm^−1^, 0.6 Hz		140	By spin coating (t = 5 µm); Ericsson cycle ^[^ ^122^ ^]^
	BTO thin film	Δ*T*: 20 to 120°C, *ΔE*: 100 to 125 kVcm^−1^, 3 kHz		30 000	By pulsed‐laser deposition (t = 200 nm); Ericsson cycle ^[^ ^123^ ^]^
	PMNT thin film	Δ*T*: 56 K, Δ*E*: 267 kVcm^−1^, 1 kHz		526 000	By pulsed‐laser deposition (t = 150 nm); Ericsson cycle ^[^ ^124^ ^]^
Optical Energy/ Photovoltaic	KNB‐NNO ceramic	Laser beam, 9.95 W cm^−2^	0.0033		t = 100 µm; V: 0.11 V, *I*: 0.03 µA cm^−2^; *η*: 0.12% ^[^ ^125^ ^]^
	KNO–BNNO thick film	Halogen lamp, 4 mW cm^−2^	0.14		t = 20 µm; V: 3.5 V, *I*: 0.04 µA cm^−2^; *η*: 3% ^[^ ^92^ ^]^
	BFO thin film	Xenon lamp, 750 mW cm^−2^	1.1		By sputtering (t = 170 nm); V: 0.28 V, *I*: 0.4 µA cm^−2 [^ ^95^ ^]^
	PLZT thin film	UV, 0.0587 mW cm^−2^	1.62		Epitaxial growth on Nb‐SrTiO_3_ (t = 100 nm); V: 0.71 (V), *I*: 2.324 (µA cm^−2^); *η*: 0.28% ^[^ ^89^ ^]^
	BTO thin film	LED, 500 mW cm^−2^	5.85		Epitaxial growth on MgO (t = 50 nm); V: 0.65 V, *I*: 9 µA cm^−2^; *η*: 1% ^[^ ^93^ ^]^
	ZnO/BFO thin film	Xenon lamp, 22.3 mW cm^−2^	74.8		By sputtering (t = 300 nm); V: 0.22 V, *I*: 340 µA cm^−2^; *η*: 0.33% ^[^ ^100^ ^]^
	BFCO thin film	Red laser, 1.5 MW cm^−2^	594		Epitaxial growth on SrTiO_3_ (t = 125 nm); V: 0.6 V, *I*: 990 µA cm^−2^; *η*: 6% ^[^ ^96^ ^]^
	BFMNO thin film	Hernia light, 110 mW cm^−2^	348.5		By spin coating (t = 400 nm); V: 5.41 V, *I*: 71.07 µA cm^−2 [^ ^97^ ^]^
	P(VDF‐TrFE) thin film	Xenon lamp, 100 mW cm^−2^	9108		Langmuir Blodgett (t = 10 nm); V: 0.66 V; *I*: 13 800 µA cm^−2^; *η*: 4.9% ^[^ ^99^ ^]^
	BTO single crystal	Xenon lamp, 470 mW cm^−2^	7140		Commercial (t = 1 mm); V: 0.42 V, *I*: 17 000 µA cm^−2^; *η*: 3.9% ^[^ ^94^ ^]^

PZT: Pb(Zr,Ti)O_3_; PZnT‐NT: PbZn_0.3_ Ti_0.7_ O_3_‐Na_2_TiO_3_; PNNZT: (Ni, Nb)‐modified PZT; PMNT: Pb(Mg_1/3_ Nb_2/3_)O_3_‐PbTiO_3_; PLZT: La‐doped PZT; BTO: BaTiO_3_; BFO: BiFeO_3_; BFCO: Cr‐modified BFO; BFMNO: (Mo,Ni)‐modified BFO; KNN: (K, Na) NbO_3_; KNFN: Fe_2_O_3_‐KNN; KNB‐NNO: KNaBa‐NbNiO; KNO‐BNNO: KNbO_3_‐BaNiNbO_3_; PVDF: polyvinylidene fluoride, P(VDF‐TrFE): poly(vinylidene fluoride‐co‐trifluoroethylene); P(VDF‐HFP): poly(vinylidene fluoride‐hexafluoropropylene)

## 4 Ferroelectrics for Energy and Data Storage

### 4.1 Electrical Energy Storage with High Permittivity

As dielectrics with the ability of directly storing electrical charge and energy, ferroelectric materials possess high nonlinear dielectric permittivity ε = *ε*_r_ε_0_ (*ε_r_*: dielectric constant, and *ε_0_*_:_ permittivity of vacuum), attributed to their highly responsive polarization to external electrical field. A dielectric capacitor, as the basic electric charge storage component made up of a dielectric medium with dielectric constant *ε_r_*, can store *ε_r_* times of charges than vacuum. The *ε_r_* of a ferroelectric material is not a real constant as it strongly depends on electrical field, and could be a few orders of the magnitude higher than that of a normal linear dielectric material. Thus ferroelectrics are highly competitive in providing high density electrical charge and electrical energy storage function.^[^ ^126^ ^]^ While electrochemical supercapacitors have much larger capacitance value, they are characterized of slower charge/discharge rate (∼sec), limited cycling life (∼10^5^) and higher leakage current (∼mA). In contrast, ferroelectric capacitors exhibit higher charge/discharge rate (∼µs to ms), almost unlimited cycling life (without requiring domain switch), and lower leakage current (∼0.1 mA). In addition, the use of electrolyte cells in supercapacitors usually limits the maximum working voltage, typically below 3.0 V, while ferroelectric capacitors can operate in hundreds of volts. The distinct characteristics of ferroelectric capacitors and electrochemical supercapacitors are attributed to their different charge storage mechanisms, which are analyzed in our previous study.^[^ ^127^ ^]^

**Figure 3:**
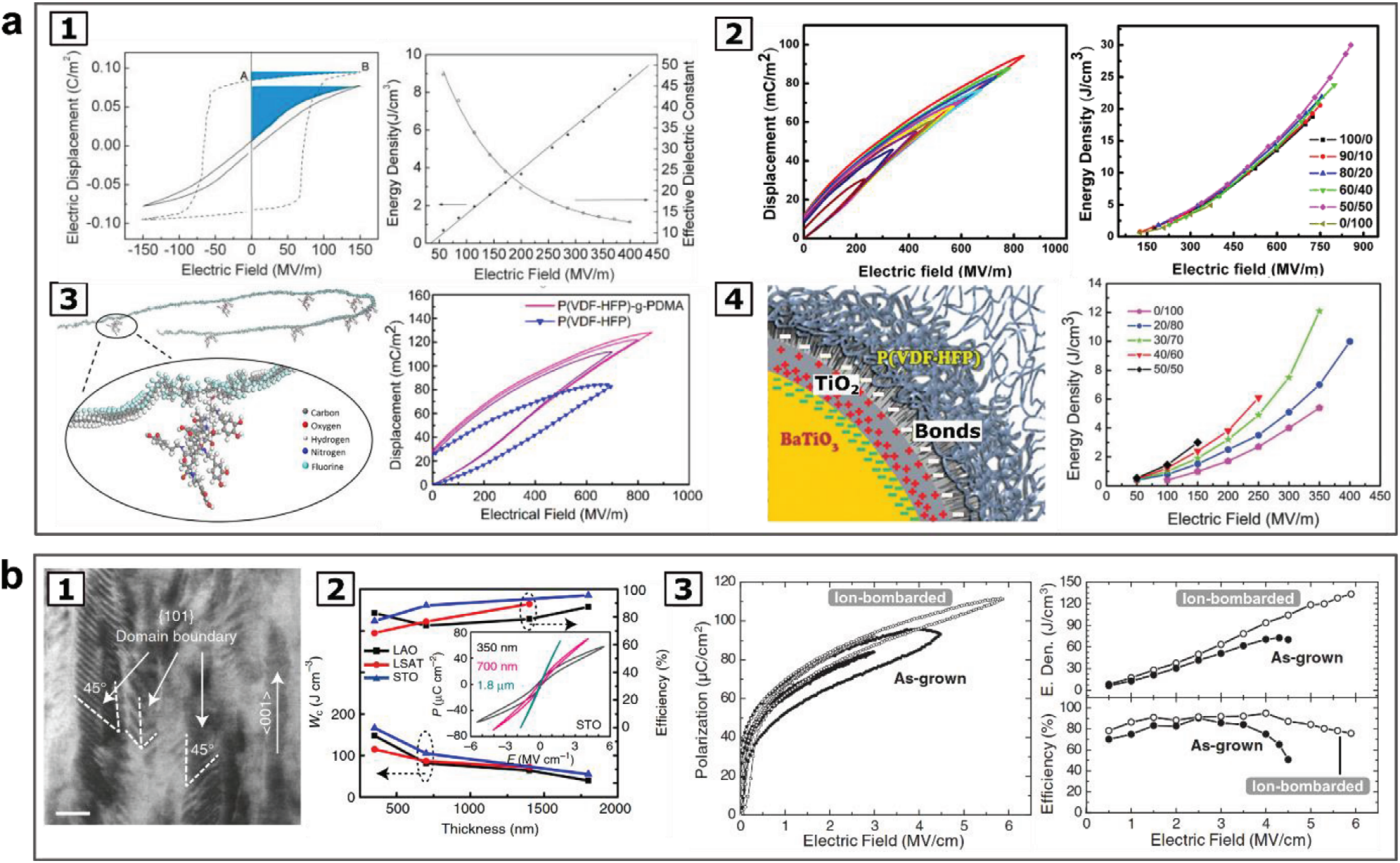
Ferroelectric materials for electrical energy storage. a) Electrical charge storage with ferroelectric polymer‐based materials: a1) Left: comparison of electric displacement–electric field (D‐E) hysteresis loops of poly(vinylidene fluoride‐trifluoroethylene) (P(VDF‐TrFE)) (VDF/TrFE = 75/25) (dotted line) and poly(vinylidene fluoride‐trifluoroethylene‐chlorofluoroethylene)) (P(VDF‐TrFE‐CFE)) (VDF/TrFE/CFE = 58.3/34.2/7.5) (solid line). The energy density is illustrated in the shaded area. Right: electric field dependance of the energy density and effective dielectric constant of the P(VDF‐TrFE‐CFE), showing the electric energy density > 9 J cm^−3^ at electric field of 400 MVm^−1^. Reproduced with permission.^[^ ^128^ ^]^ Copyright 2006, The American Association for the Advancement of Science. a2) Left: displacement–electric field hysteresis loops of P(VDF‐HFP)/PVDF (50/50). Right: energy density for the P(VDF‐HFP)/PVDF with different compositions. Reproduced with permission.^[^ ^130^ ^]^ Copyright 2012, AIP Publishing. a3) Left: illustration of a P(VDF‐HFP) copolymer chain grafted with PDMA side chains. Right: displacement–electric field hysteresis loops of P(VDF‐HFP)‐g‐PDMA film, in comparison to the hysteresis loop of P(VDF‐HFP) film. Reproduced with permission.^[^ ^131^ ^]^ Copyright 2013, AIP Publishing. a4) Left: diagram showing the cross‐sectional structure of the core–shell nanoparticles of BaTiO_3_@TiO_2_ in the matrix of P(VDF‐HFP). Right: energy density of the P(VDF‐HFP)‐BaTiO_3_@TiO_2_ nanocomposites of different compositions.^[^ ^137^ ^]^ Reproduced by permission of the PCCP Owner Societies, 2013. b) Electrical charge storage with ferroelectric ceramic‐based materials: b1) Cross‐section of a 0.7‐µm‐thick Ba(Zr,Ti)O_3_ film (scale bar: 20 nm), showing second‐order “nano‐domains” with (101) boundaries. b2) Energy storage densities and efficiencies (*W*_C_ and *η*) of the Ba(Zr,Ti)O_3_ films on different substrates in different thicknesses; inset: typical polarization‐electric field hysterisis loops. Reproduced from Reference^[^ ^143^ ^]^] under the terms of the CC‐BY Creative Commons Attribution 4.0 International License (https://creativecommons.org/licenses/by/4.0) (CC BY 4.0), 2017. b3) High‐field energy storage performance of relaxor ferroelectric thin films made of relaxor Pb(Mg_1/3_Nb_2/3_)O_3_–PbTiO_3_ (PMN‐PT) with and without high‐energy ion‐bombardment: Left: comparison of hysteresis loops of as grown film and the film after high‐energy ion‐bombardment; Right: electric field dependance of the energy density and energy strorage efficiency of the films calculated from unipolar hysteresis loops, showing an ultrahigh energy density of 133 J cm^−3^ with efficiencies above 75%. Reproduced with permission.^[^ ^144^ ^]^ Copyright 2020, The American Association for the Advancement of Science.

Many progresses have been made in the design and fabrication of various ferroelectric polymers,^[^ ^128^, ^129^, ^130^, ^131^ ^]^ ceramics,^[^ ^132^, ^133^, ^134^, ^135^ ^]^ composites,^[^ ^136^, ^137^, ^138^ ^]^ multilayers,^[^ ^138^, ^139^ ^]^ and antiferroelectrics,^[^ ^140^, ^141^, ^142^ ^]^ with improved performance for high power energy storage uses, with several examples given in **Figure** **3**. Figure 3a introduces several ferroelectric polymers with high energy density and fast discharge speed, and Figure 3b presents an outstanding energy density up to the order of 10^2^ J cm^−3^, as reported in ferroelectric thin layers of sub‐micrometer thickness, with higher coercive field and breakdown field than the bulk counterpart.^[^ ^143^, ^144^ ^]^

Since the energy storage density is lower in orders of magnitude than electrochemical supercapacitors and further lower than batteries, in which chemical energy storage is involved, ferroelectric materials are typically not competitive for general massive energy storage today. However, the much higher power density of ferroelectric energy storage due to the extremely high direct electrical charging and discharge rate is valuable for applications of insulated‐gate bipolar transistor snubbers, high‐frequency inverters, pulsed power generation and power factor correction,^[^ ^145^ ^]^ and thus is attracting great attention and research efforts with the rapid growth of electrified vehicles and systems. The ability of generating high power electrical signals in pulsed mode, in conjunction with high rate and durable charging and discharging cycling, are desired for storing and transmitting the electrical outputs for distributed self‐powered wireless sensors and transducers.

### 4.2 Ferroelectric Data Storage and Nonvolatile Memories

Ferroelectric materials have been used to produce three main types of memories: capacitor‐based, transistor‐based, and resistor‐based memories. In principle, the high permittivity of ferroelectric thin films is attractive as the dielectric medium for high density capacitor to store electrical charge for realizing dynamic random access memory (DRAM) without utilizing any switchable polarization states. In this case, the information stored in DRAM is volatile due to the loss of electrical charge with leakage, and thus periodical refreshment of charges is required for the memory function, which constantly consumes electric power.^[^ ^146^, ^147^ ^]^ However, the application of ferroelectrics in DRAM is not practically competitive due to the issues of small size/thickness degradation, leakage, and relatively low‐density integration. In the literature, there are ongoing research efforts to explore oxide ultrathin films with simplified compositions and improved downsize scalability for DRAM application.^[^ ^148^ ^]^

To realize a nonvolatile memory, the two spontaneous polarization states of ferroelectric thin films, which are switchable by an external bias above the coercive field, are utilized to produce ferroelectric random access memories (FeRAM) that has the two corresponding stable states with a long polarization retention time.^[^ ^149^, ^150^ ^]^ With progresses achieved during 1990s to 2000s, fast FeRAM with high endurance cycles (up to 10^12^–10^14^) is achieved.^[^ ^151^, ^152^ ^]^ While FeRAM has relatively lower integration density compared to alternative nonvolatile memory technologies, such as spin‐transfer torque random‐access memory (STT‐RAM), phase‐change memory (PCM), magnetic random‐access memory (MRAM), it has good application values in small consumer devices such as handheld phones, personal digital assistants (PDAs), smart cards, power meters, and in security systems.^[^ ^153^ ^]^

For FeRAM, a major disadvantage is the state‐destructive read out operation. It involves applying a bias above the coercive field of the ferroelectric thin film for obtaining the state data by detecting if there is a current pulse accompanied by polarization switching corresponding to the Off state, which requires relatively large applied voltage and energy consumption. This destructive read out operation with write power consumption of FeRAM can be overcome by constructing a field‐effect transistor memory using the ferroelectric film as the gate (FeFET), which can be read out with the source‐drain current controlled by the ferroelectric polarization in the gate. The combination of the three terminal transistor configuration and bi‐stable spontaneous polarization states make it feasible to achieve extremely low power consumption on FeFET, which can reach ≈1 fJ bit^−1^.^[^ ^154^, ^155^, ^156^ ^]^

**Figure 4:**
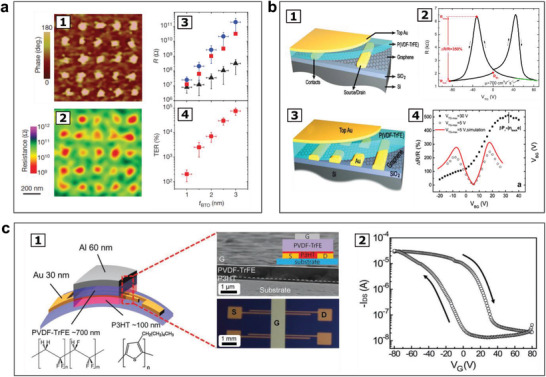
Data storage and nonvolatile memory devices made of ferroelectric materials. a) Ferroelectricity of nanometer‐thick highly strained BaTiO_3_ films and demonstration of nondestructive reading of the polarization state of the BaTiO_3_ film using tunnelling current: a1) Piezoresponse force microscopic phase images of a 70 nm‐in‐diameter dots in 5 × 5 matrix with distance of 200 nm on a 2 nm‐thick BaTiO_3_ thin film. a2) Resistance maps of the BaTiO_3_ thin film obtained by conductive‐tip atomic force microscopy, in which a significant difference in resistance was observed between the background and the dots corresponding to the two resistance states. a3) The relationship of BaTiO_3_ thickness with the resistance (R) (Red squares: unpoled region; Black triangles: positively poled region; Blue circles: negatively poled region), showing an exponential increase of resistance with increasing the BaTiO_3_ thickness, as expected for direct tunnelling. a4) Exponential increase of tunnelling electro‐resistance (TE) with increasing BaTiO_3_ thickness (75 000% for the 3 nm‐thick BaTiO_3_ film). Reproduced with permission.^[^ ^159^ ^]^ Copyright 2009, Springer Nature. b) A nonvolatile ferroelectric FeFET memory made of ferroelectric polymer P(VDF‐TrFE) on graphene: b1) Schematic illustration of the nonvolatile memory device made of graphene field‐effect transistor using ferroelectric gating. b2) Resistance change ratio Δ*R*/*R* of 350% in resposne to the gate voltage. Reproduced with permission.^[^ ^162^ ^]^ Copyright 2009, AIP Publishing. b3) Diagram of another nonvolatile memory device made of graphene‐based field‐effect transistor using ferroelectric gating with improved design. b4) By increasing the maximum top gate voltage (*V*_TGmax_) from 5 to 30 V, the maximum Δ*R*/*R* was increased to 500%. Reproduced with permission.^[^ ^164^ ^]^ Copyright 2010, American Physical Society. c) Organic FeFET on flexible substrate: c1) Schematic illustration of a top‐gate bottom‐contact (TGBC) FeFET memory, comprising a poly(3‐hexyl thiophene) (P3HT) channel and ferroelectric insulator made of P(VDF‐TrFE), with microscopic images of the surface and cross‐section for the device. c2) Drain‐current/gate voltage (*I*_DS_ − *V*_G_) curve of the device, wherein a acharacteristic current hysteresis was observed due to the nonvolatile polarization of the ferroelectric P(VDF‐TrFE) film. Reproduced with permission.^[^ ^166^ ^]^ Copyright 2012, Wiley VCH.

Since the polarization orientation of the ferroelectric film sandwiched between two electrodes changes the carrier‐depletion width at interfacial energy barriers, and thus the conductivity of delocalized electrons or conducting tunneling probability for an ferroelectric ultrathin film,^[^ ^157^, ^158^ ^]^ the electrode‐sandwiched ferroelectric structure exhibits switchable resistive state to function as a resistor‐based nonvolatile memories with further simplified configuration and miniaturization.^[^ ^159^, ^160^ ^]^ In the study as presented in **Figure** **4**a, nondestructive reading of the polarization state was demonstrated using tunneling current on highly strained nanometer‐thick ferroelectric BaTiO_3_ films. Besides the primary contributions from interfacial energy barrier on the conductance switching, it was noted that the domain wall configurations and the related polarization discontinuities could also affect the conductance,^[^ ^161^ ^]^ which is explored for potential multistate neuromorphic computing applications.

Nonvolatile ferroelectric memories including organic FeFET were also demonstrated from ferroelectric polymer materials, including P(VDF‐TrFE) on graphene and flexible substrates through solution and printing process, as illustrated in Figure 4b,c, respectively.^[^ ^162^, ^163^, ^164^, ^165^, ^166^ ^]^ With the advancement in low cost, large area printing and coating fabrication techniques such as screen printing, spray coating and inkjet printing, mass production of organic nonvolatile ferroelectric memories are expected to be realized for the future flexible electronics.

These characteristics of ultralow power consumption, multistate neuromorphic computing feature, and compatibility with printable process of nonvolatile memories enabled by various ferroelectric materials are desired for energy autonomous system applications.

## 5 Synergized Multiple Ferroelectric Functions for Distributed Intelligence

### 5.1 Devices Enabled with Multiple Ferroelectric Functions

**Figure 5:**
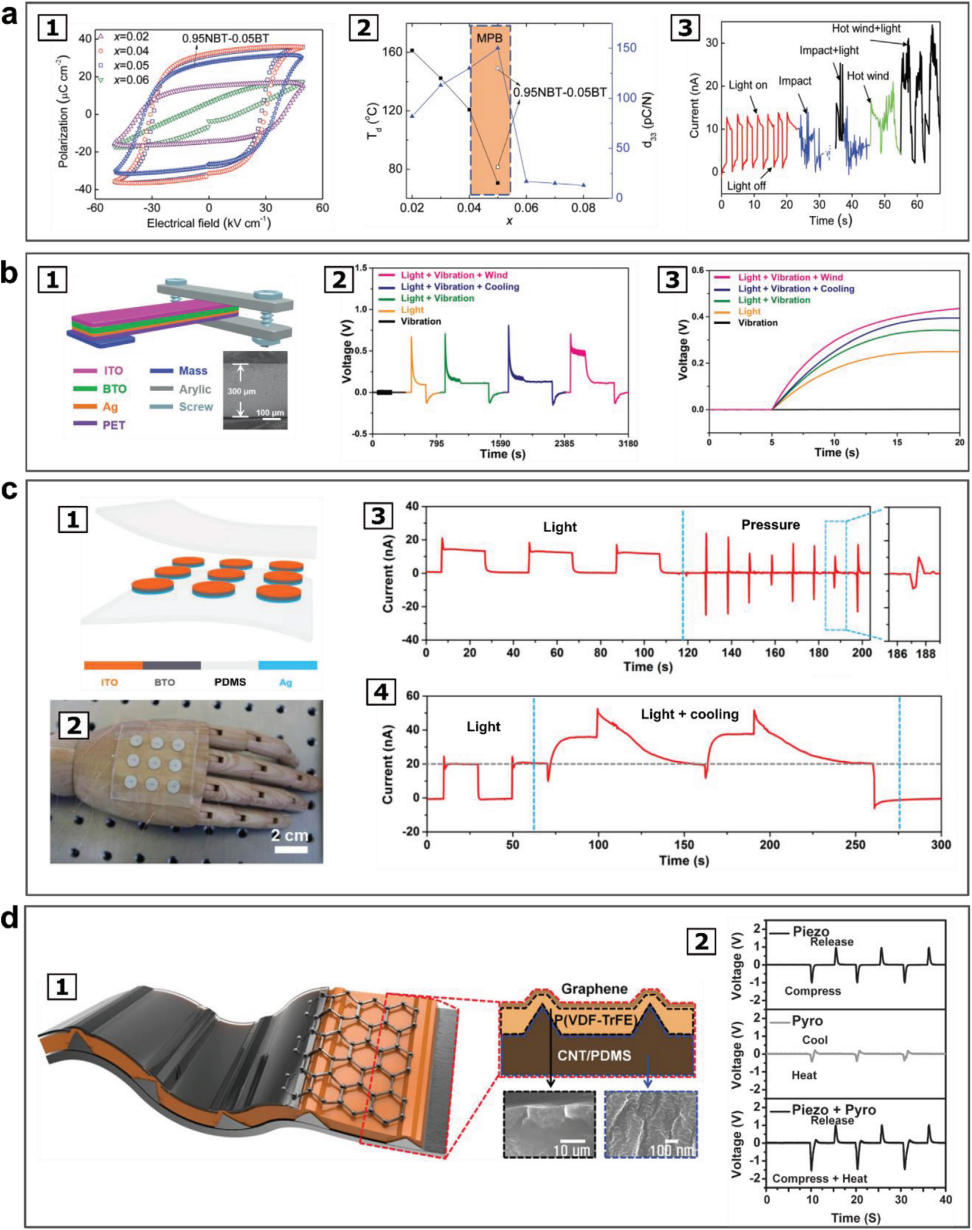
Devices enabled with multiple ferroelectric functions. a) Multifunctional ferroelectric and photovoltaic material made of (1−*x*) (Na,Bi)TiO_3_‐xBa(Ti,Ni)O_3_: a1) P‐E hysteresis loops of (1−*x*)(Na,Bi)TiO_3_‐xBa(Ti,Ni)O_3_ ceramics of different compositions, showing saturated hysteresis loop with high remnant polarization with the composition of *x* = 0.04 and 0.05 in the MPB region. a2) Change of the depolarization temperature (*T*_d_) and piezoelectric coefficient (d_33_) with the *x* in (1−*x*)(Na,Bi)TiO_3_‐xBa(Ti,Ni)O_3_. The rectangular area shows the MPB region. a3) Multisignal response current of 0.95(Na,Bi)TiO_3_‐0.05Ba(Ti,Ni)O_3_ in response to input energy of light, impact, and hot wind. Reproduced with permission.^[^ ^168^ ^]^ Copyright 2019, Wiley VCH. b) Increasing charge quantity in ferroelectric BaTiO_3_ by coupling the effects of piezo–pyro–photoelectricity: b1) Schematic illustration of the BaTiO_3_‐based energy harvestor; inset: cross‐sectional image of the BaTiO_3_ film. b2) Output voltage of the BaTiO_3_‐based energy harvesting device in different conditions. b3) Performance of the energy harvestor for charging a capacitor (4.7 µF) at different conditions. Reproduced with permission.^[^ ^171^ ^]^ Copyright 2019, RSC Publishing. c) Ferroelectric BaTiO_3_‐based multifunctional nanogenerator for harvesting optical, mechanical and thermal energies: c1) Structure diagram of the nanogenerator. c2) Photo of the BaTiO_3_‐based multifunctional nanogenerator attached to a prosthetic hand. c3) Output current signals of the nanogenerator under illumination (wavelenght: 405 nm) and finger pressure. c4) Output current of the nanogenerator under different conditions: illumination only versus simultaneous illumination and cooling. Reproduced with permission.^[^ ^172^ ^]^ Copyright 2020, Elsevier. d) Ferroelectric P(VDF/TrFE) film‐based stretchable hybrid nanogenerator for harvesting mechanical energy and thermal energy: d1) Structure diagram of the nanogenerator and the cross‐sectional morphology. d2) Output voltage of the nanogenerator in different conditions. Reproduced with permission.^[^ ^175^ ^]^ Copyright 2014, Wiley VCH.

By combining the multiple functions as described above, ferroelectric materials exhibit the great values for enabling various novel devices. While ferroelectric materials can convert kinetic, thermal and optical energy into electricity with their piezoelectric, pyroelectric, photovoltaic and photochemical properties, respectively,^[^ ^167^ ^]^ it is not common for one single ferroelectric material to be a high performer in all the energy conversion effects. For example, for achieving a ferroelectric material with both large piezoelectric effect and photovoltaic current, one challenge to overcome is to obtain a ferroelectric semiconductor that can maintain strong piezoelectric effect. For producing a large photovoltaic current response under visible light, a low energy bandgap is required, but such a low energy bandgap often leads to electrically leaky ferroelectric material that generally degrades piezoelectricity. One method for simultaneously achieving high piezoelectricity and low bandgap was to introduce gap states at ferroelectric morphotropic phase boundary (MPB) where the local polar heterogeneities induced by the defects were coupled with the host polarization. As illustrated in **Figure** **5**a, this was demonstrated in (1−*x*)Na_0.5_Bi_0.5_TiO_3_‐xBa(Ti_0.5_Ni_0.5_)O_3−*δ*_ with a low bandgap of 0.9 eV (*x* = 0.02‐0.08, Ni^2+^‐mediated), showing visible/near‐infrared light absorption and excellent piezoelectricity at room temperature. With MPB at *x* = 0.05, the material showed the effective piezoelectric coefficient *d_33_* of 151 pCN^−1^, *P_r_* of 31.2 μCcm^−2^, and a photocurrent density about two orders of magnitude higher than ferroelectric (Pb,La)(Zr,Ti)O_3_.^[^ ^168^ ^]^

In another (1−*x*)(K_0.48_Na_0.52_)NbO_3_‐*x*(Bi_0.5_Na_0.5_)(Zr_0.55_Ni_0.45_)O_3−*δ*_ (KNN‐BNZN) ceramic system with narrow bandgap, a large piezoelectric coefficient *d_33_* of about 318 pCN^−1^ was obtained at *x* = 0.04. A rhombohedral‐orthorhombic‐tetragonal (R‐O‐T) phase boundary was observed, showing the role of defect dipoles (Ni^2+^‐V_o_^2−^) for sustaining the strong piezoelectric and ferroelectric properties at MPB region. Furthermore, a small energy bandgap ∼2.5 eV could be obtained (*x* > 0.02) with improved photovoltaic response under AM 1.5 irradiation. The large near‐infrared (NIR) photoresponse with current density of ∼100 nA cm^−2^ was promising for NIR devices.^[^ ^169^ ^]^ These results show the potential of obtaining piezoelectric and visible/NIR responsive ferroelectric oxides for solar energy conversion, NIR detection, and electromechanical multifunctional applications.

In another example, a PZT film deposited on Pt/Ti/SiO_2_/Si substrate provided a large recoverable dielectric energy storage density of ∼11.2 J cm^−3^ with energy utilization efficiency ∼68%. Additionally, the film exhibited a significant photovoltaic effect, with an open circuit voltage and a short circuit current of −1.01 V and 155 µAcm^−2^, respectively.^[^ ^170^ ^]^ The authors claimed their results from the ferroelectric PZT films provided potential bi‐functional applications on energy storage and photovoltaic effect, although further efforts are required to turn the bi‐functional behaviors into a practical application case.

In one example as illustrated in Figure 5b, Y. Je et al demonstrated enhanced electrical charge and energy generation in ferroelectric BaTiO_3_ by forming constructive multiple piezoelectric, pyroelectric, and photovoltaic effects, when the photovoltaic effect was investigated under the combination of various external stimuli, including vibration, wind, light, and cooling. A temperature increase caused by light illumination was observed, while a temperature decrease under wind, vibration, and cooling process. The *d_33_* value increased with decreasing temperature, decelerating electric dipole oscillation, which improved the photovoltaic performance induced by the depolarization field. The device exhibited the constructive electrical charge and energy generation performance under operation conditions of “‘light + vibration + wind”’, and a capacitor (4.7 µF) was charged to 0.44 V within 15 s.^[^ ^171^ ^]^ The recent progress by the same group showed a much larger electrical output obtained from the coupled multifunctional effects than the individual piezoelectric, photovoltaic, and pyroelectric effects, as presented in Figure 5c. Under intensity of ≈83.2 mWcm^−2^, the current under simultaneous light illumination and pressure (7.6 kPa) was enhanced by ≈387.3% over that under light illumination only. By cooling with Δ*T* = −19.5 K, the output current was improved by 375.0% under a light intensity of 83.2 mWcm^−2^. When the flexible sensor array system was mounted on a prosthetic hand, detection of the distribution of pressure, light, and temperature variations was demonstrated without separated power source in principle.^[^ ^172^ ^]^ Although a really practical self‐powered sensor system was not demonstrated in the work, these results indicated the feasibility for simultaneous energy scavenging of light, thermal and kinetic energies, or the possibility of detecting multiple parameters by collectively utilizing photovoltaic, pyroelectric and piezoelectric functions of a ferroelectric material.

With low temperature processing, flexibility, and convenience to be produced in various forms from coating, foil to fiber, ferroelectric polymers have been demonstrated for many types of the multimode energy harvesters and sensors. A review well conducted by B. Stadlober et al showed the potential of the multifunctionalities of ferroelectric polymers, particularly PVDF, as prime candidates for monitoring diverse mechanical, thermal and vital parameters, and integration in multifaceted electronics and sensor devices.^[^ ^173^ ^]^

J. Park et al demonstrated multiple functionalities of e‐skins for enabling spatiotemporal recognition of multiple static and dynamic tactile stimuli. The temperature, pressure, and vibration could be detected by pyroelectric, piezoelectric, and piezoresistive sensing capabilities of ferroelectric composite films comprising reduced graphene oxide and PVDF. The temperature‐dependent pressure monitoring of artery vessels, high‐precision acoustic sound detection, and texture recognition of varied surfaces were exhibited through experimental work. The results suggested the capabilities of ferroelectric multifunctions of providing an e‐skin platform for realizing humanoid robotics and wearable medical diagnostic systems.^[^ ^174^ ^]^ In another example, as illustrated in Figure 5d, J.‐H. Lee et al demonstrated flexible and stretchable hybrid energy harvesting device based on a micro‐patterned P(VDF‐TrFE) piezoelectric film, which was able to harvest both thermal and mechanical energies under various modes of applied thermal gradient and strain, as pyroelectric and piezoelectric power outputs.^[^ ^175^ ^]^

These energy harvesting functions can be combined with organic sensor and memory functions in ferroelectric polymers, which exhibits the potential application values for health monitoring, motion detection and electronic skin.^[^ ^176^ ^]^

### 5.2 Energy Autonomous Wireless Sensors with Synergized Ferroelectric Functions

#### 5.2.1 Battery‐Less Sensors Powered by Separate Energy Harvester

Batteries often provide a convenient and versatile power source for wireless sensor systems, particularly when there is no mass or size restriction, or the projected lifetime of the sensor node is no more than a few years. As an alternative approach, harvesting energy available in the environment offers another attractive power solution for distributed wireless sensor systems, particularly for implementation of a large number of miniaturized sensors or without any expected maintenance over a long operation lifetime, such as for long term monitoring.^[^ ^177^ ^]^ Furthermore, in some cases, a hybrid system comprising both energy harvesters and rechargeable batteries could be more competitive as a practical solution. It has been well analyzed that harvesting kinetic energy with piezoelectric effects of ferroelectric materials can feasibly provide power for low duty cycle of wireless sensor nodes, with advantages in term of energy density over capacitive converters and without requiring separate voltage source.^[^ ^178^ ^]^

**Figure 6:**
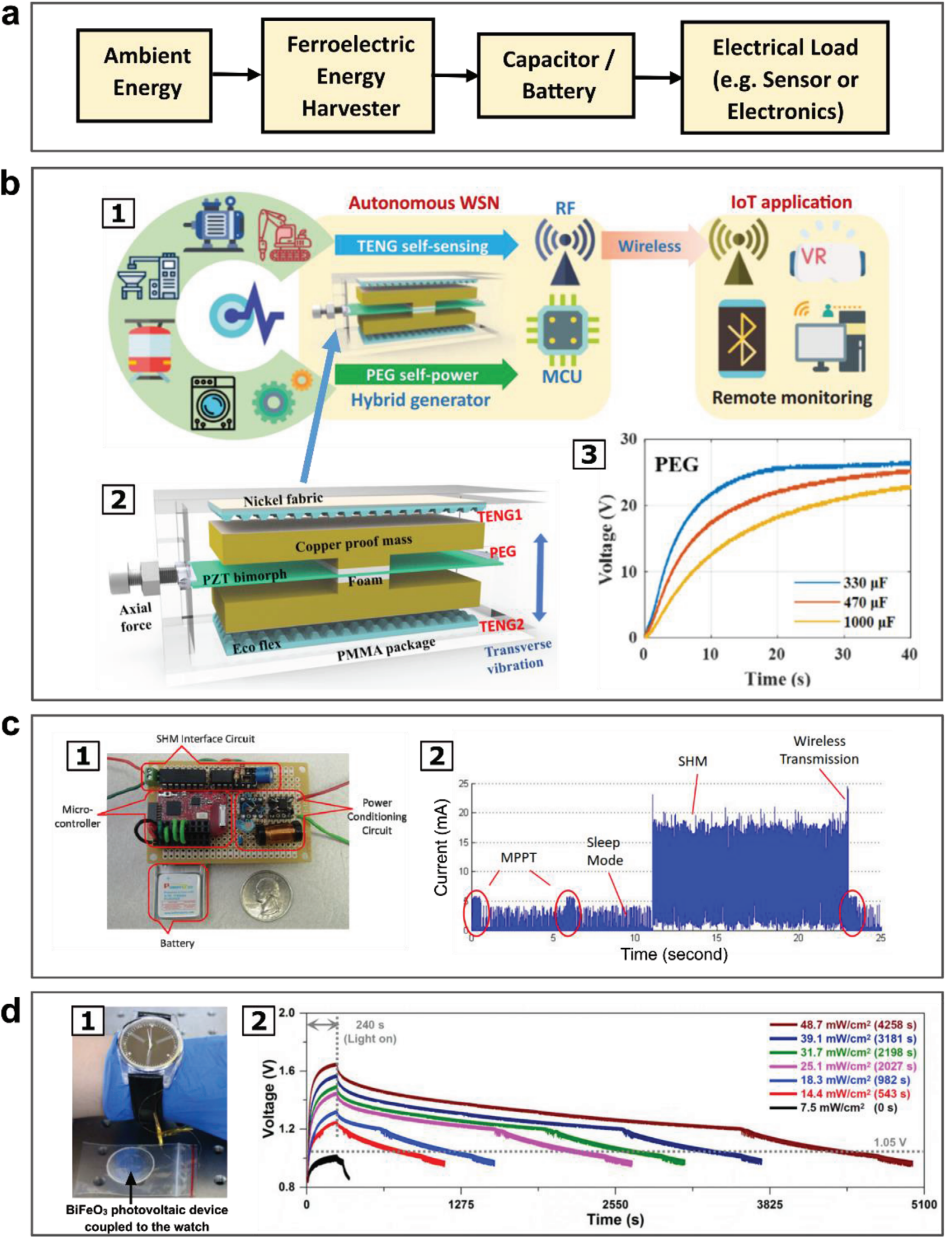
Self‐powered systems driven by ferroelectric transducers dedicated for power generation under ambient excitations. a) Conventional architecture of a self‐powered system consisting of a ferroelectric harvester coupled to a capacitor or battery powering up the electrical load of a sensor or electronic device. b) An autonomous wireless sensing system driven by a hybrid device consisting of a piezoelectric energy generator (PEG) implemented on a PZT bimorph and triboelectric generator (TENG) functioning as the sensors: b1) Concept illustration of the implementation of PEG‐TENG hybrid device for self‐powered wireless IoT applications. b2) Detailed architecture of PEG‐TENG hybrid device. b3) Piezoelectric generator charging up capacitors to high voltages above 20 V. Reproduced with permission.^[^ ^179^ ^]^ Copyright 2021, Elsevier. c) A wireless sensor for structural health monitoring (SHM) driven by a piezoelectric cantilever: c1) A photo of the self‐powered SHM sensing node prototype. c2) Current consumption profile of the self‐powered sensing node at different phases of operation: MPPT (Maximum Power Point Tracking), sleep, SHM sensing and wireless transmission. Reproduced with permission.^[^ ^180^ ^]^ Copyright 2010, SPIE. d) Self‐powered electronic watch sustained by BiFeO_3_ photovoltaic device as the energy harvester: d1) A photo of the electronic watch coupled to BiFeO_3_ photovoltaic device serving as the power source. d2) Voltages across the electronic watch after light‐off sustained by the energy in Li‐ion battery charged up earlier by the BiFeO_3_ photovoltaic device exposed to light with different intensities. Reproduced with permission.^[^ ^182^ ^]^ Copyright 2019, Elsevier.

The use of ferroelectric materials as energy harvesters to generate sustainable power for realizing energy autonomous devices is further explained with examples as shown in **Figure** **6**. A general architecture of a typical self‐powered system with a separate ferroelectric energy harvester is illustrated in Figure 6a. In Figure 6b, an energy autonomous wireless sensing node developed by Wang et al was driven by a hybrid device consisting of a PZT bimorph piezoelectric generator (PEG) as the power source and triboelectric nanogenerator (TENG) as the acceleration sensor.^[^ ^179^ ^]^ The PEG power source achieved 6.5 mW under the acceleration magnitude of 1.0 g at 25 Hz, and was able to sufficiently sustain the wireless sensing node's electronics, inclusive of the microcontroller system and RF transceiver, to transmit the acceleration data acquired by the TENG sensor wirelessly by Zigbee.

In Figure 6c, a self‐powered impedance‐based sensor node without battery was used for wireless structural health monitoring (SHM) with ability to detect local damage efficiently. The system was powered by a piezoelectric PZT cantilever for harvesting kinetic energy, which could obtain 2.9 mW under 0.5 g acceleration. It was demonstrated that the energy harvested was sufficient to operate in an intermittent SHM mode once every two minutes.^[^ ^180^ ^]^ Wireless node powered by a separate nonlinear piezoelectric bistable energy harvester was demonstrated by harvesting energy from low frequency vibration for transmitting data at 2.4 GHz without any battery, promising for scavenging energy from wideband vibrations in the environment for wireless signal transmission.^[^ ^181^ ^]^

Y. Ji et al demonstrated an electronic watch powered by photovoltaic output from ferroelectric BiFeO_3_ (BFO), as illustrated in Figure 6d. After photocharged for 240 s by the photovoltaic device made from BFO, a capacitor of 3300 µF was able to sustain the continuous operation of an electronic watch for 1785 s. A rechargeable battery was also used to extend the operation time of the watch to 4258 s continuously. The results showed a good example for realizing ferroelectric‐based photovoltaic devices with large output voltages for self‐powered electronics.^[^ ^182^ ^]^

While a portable and wearable self‐powered system is a promising type of human–machine interaction interface, many existing problems should be addressed before flexible self‐powered system can move to the stage of large‐scale practical applications, including performance degradation during long‐term operation, lack of standardized manufacturing process and operation schemes.^[^ ^183^ ^]^ For many portable and wearable self‐powered electronic devices, power management technologies are demanded to work compatibly with the electrical outputs with large variation in frequency, amplitude and waveform, and even power unavailability during certain intervals due to the uncertainty of the energy source in the ambient. Reliable wireless operation module is desired to realize the noncontact signal transmission without wire connection. Ferroelectric functions are promising for providing the technical solutions as illustrated with the examples and the analyses here.

#### 5.2.2 Self‐Powered Sensor Concepts without Any Separate Energy Source

In Section 2.1, it has been emphasized that ferroelectric materials possess the in‐principle self‐powered sensing mechanisms. Indeed, many ferroelectric sensors with the ability to simultaneously harvest energy and detect stimuli have been reported. However, the demonstration are often for proving the sensor device concept, but not at a practically working system level. Y. Yang's group demonstrated a photovoltaic‐pyroelectric system with ferroelectric BaTiO_3_ sheets that are capable of simultaneous sensing and energy harvesting functions. With simultaneous light illumination and cooling, the photodetector system delivered improved photocurrent and photovoltage due to ferro‐pyro‐phototronic effect with ability for detection of both light illumination and temperature changes.^[^ ^184^, ^185^, ^186^ ^]^

MAPbI_3_ (Methylammonium lead iodide) is a semiconductive ferroelectric material and has ion migration effects that could form reversible p–n (or p–i–n) like structures, leading to switchable large anomalous photovoltaic effect dominated by ferroelectric polarization.^[^ ^187^ ^]^ By interfacing it with a ZnO transport layer that functioned as an electron extraction layer for improving the device stability,^[^ ^188^ ^]^ R. Saraf et al demonstrated a tactile sensor utilizing MAPbI_3_ ferroelectric and semiconducting properties and powered by solar energy.^[^ ^189^ ^]^ The ZnO nanosheets/MAPbI_3_ combinational structure formed a purely solar‐driven transistor type pressure sensor, with pressure sensitivity of 0.57 kPa^−1^, limit of detection of 0.5 kPa, and linear response over 0–76 kPa.

A self‐powered memory system to monitor and memorize in 1D and 2D motion was demonstrated by coupling ferroelectric P(VDF‐TrFE) films with a sliding triboelectric nanogenerator (TENG) and another single‐electrode TENG matrix. The memory system could record the displacement of a sliding TENG and retrieve the motion trace on the single‐electrode TENG matrix. The electroded P(VDF‐TrFE) film with a size of 3.1 mm^−2^ could memorize a minimum area changing of 30 mm^−2^, while it could work stably over a velocity range of 0.001 to 5 m s^−1^, showing the feasibility for monitoring mechanical motions without separate energy source.^[^ ^190^ ^]^

B. Andò et al recently explored the possibility to exploit one device comprising piezoelectric transducers as both a nonlinear kinetic harvester and as a sensor to detect mechanical vibrations, thus labeling the system as a fully autonomous sensor that did not require an external power source.^[^ ^191^ ^]^ In the system, the contribution to the output voltage due to the noise was used to unambiguously estimate the noise level using a thresholding and windowing algorithm. The device could operate in principle with energy autonomy in the presence of noisy vibrations superimposed on a subthreshold deterministic input signal, notwithstanding there was still use of electrical input in the experimental demonstration.

While the devices as introduced above showed the potential of ferroelectrics for self‐power operation, energy autonomous feature enabled by the material functionalities was not demonstrated at a system operation level. The experimental demonstrations mainly showed or reaffirmed the feature of ferroelectric sensing mechanisms in which the electric output is generated by directly converting the stimuli, and the nonvolatile memory function. From this perspective, even a strain sensor in the basis of piezoelectric effect could be regarded as self‐powered.^[^ ^192^ ^]^ Transmission of the electrical output from the ferroelectric sensors or memories to a working station without a physical contact is typically required for realizing a practical wireless sensor system application.

**Figure 7:**
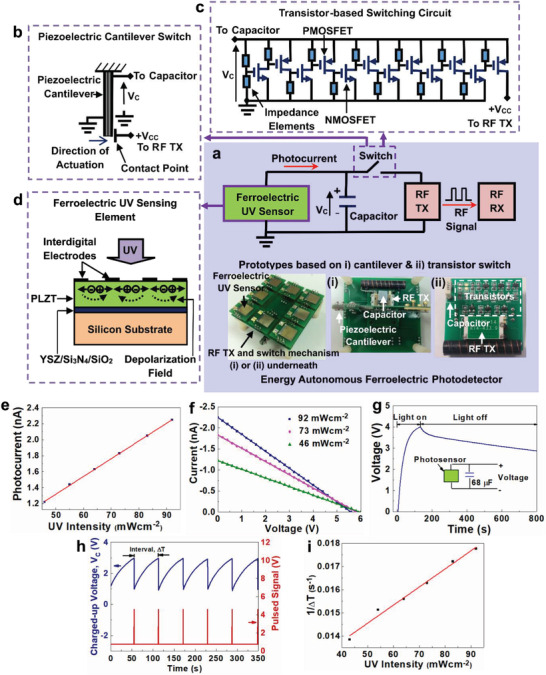
Battery‐less and wireless photo‐detector system, whereby photovoltaic sensing and energy harnessing are concurrently realized in ferroelectric thin film based on lanthanum‐doped lead zirconate titanate (PLZT). a) System architecture and prototype of the battery‐less and wireless photodetector. b) Mechanical switch based on piezoelectric cantilever driven by photovoltage implemented in the prototype. c) Electronic switch based on cascading transistors controlled by photovoltage implemented in the prototype. d) Device structure of ferroelectric photovoltaic sensing element comprising PLZT thin film with electrical polarization in the direction along the in‐plane electrodes. e) Photo‐responsivity characteristics of the ferroelectric photovoltaic sensor. f) Linear *I*–*V* characteritics of ferroelectric photovoltaic sensor delivering high photovoltage between 5.6 and 6.0 V. g) Illustration of low‐leakage characteristics of ferroelectric photovoltaic sensor enabling stored charge in a polyester capacitor to be retained in the dark. h) Charging‐discharging operation across the capacitor driven by ferroelectric photovoltaic sensor generating wireless pulses captured by RF receiver at every discharging action. i) Frequency of the RF pulses (1/Δ*T*) generated by the battery‐less photo‐detector prototype exhibits a linear relationship with UV intensity. Reproduced with permission.^[^ ^193^ ^]^ Copyright 2013, AIP Publishing.

The authors’ group proposed a self‐sustainable mechanism for simultaneously sensing and harnessing photon energy. A real battery‐less and wireless ferroelectric ultraviolet sensor was demonstrated. The prototype was made of lanthanum‐doped lead zirconate titanate (PLZT) thin film with in‐plane polarization configuration, as illustrated in **Figure** **7**. The architecture and sample are presented in Figure 7a. The concept involved accumulating and storing electrical charge from BPVE in the PLZT thin film (the ferroelectric photovoltaic UV sensing element as illustrated in Figure 7d, and its photo‐responsivity in Figure 7e), and transferring the stored charge through a switch controlled by the electrical potential of the photo‐induced charges, to a RF transmitter. The count of the RF pulses per unit time generated from the transmitter (inversely proportional to the time‐interval of the RF pulses) was proportional to the optical intensity, as exhibited in Figure 7h,i. Thus the charge storage function of the ferroelectric PLZT film with low electrical leakage, linear current‐voltage, and large bulk photovoltage characteristics of ferroelectric BPVE, as exhibited in Figure 7f,g, were combined with synergy to enable the battery‐less and wireless optical sensor operation.^[^ ^193^ ^]^

In our original work, a piezoelectric cantilever was first used as a mechanical switch driven by the photovoltage of ferroelectric BPVE to achieve the sharp On‐Off switching feature as required to minimize the leakage during the transient stage between the On and Off states, as exhibited in Figure 7b. Alternately, a ultra‐sharp switch with low leakage and constructed with cascading metal‐oxide field‐effect transistors (MOSFETs) was used later to replace the piezoelectric mechanical switch, as exhibited in Figure 7c.^[^ ^194^ ^]^ Here the optical signals, as the external stimuli to be sensed and monitored, provided the energy harnessed by the ferroelectric sensor itself and powered the wireless sensor signal transmission, without another separate energy harvester to power the sensor operation and wireless signal transmission.

With the similar operation concept, by taking use of the other ferroelectric charge‐generation sensing mechanisms, such as piezoelectric and pyroelectric, in combination with the charge storage properties, ferroelectric materials can be used to produce battery‐less and wireless electromechanical and thermal sensors, such as accelerometers and temperature sensors, respectively.^[^ ^181^, ^195^, ^196^ ^]^

**Figure 8:**
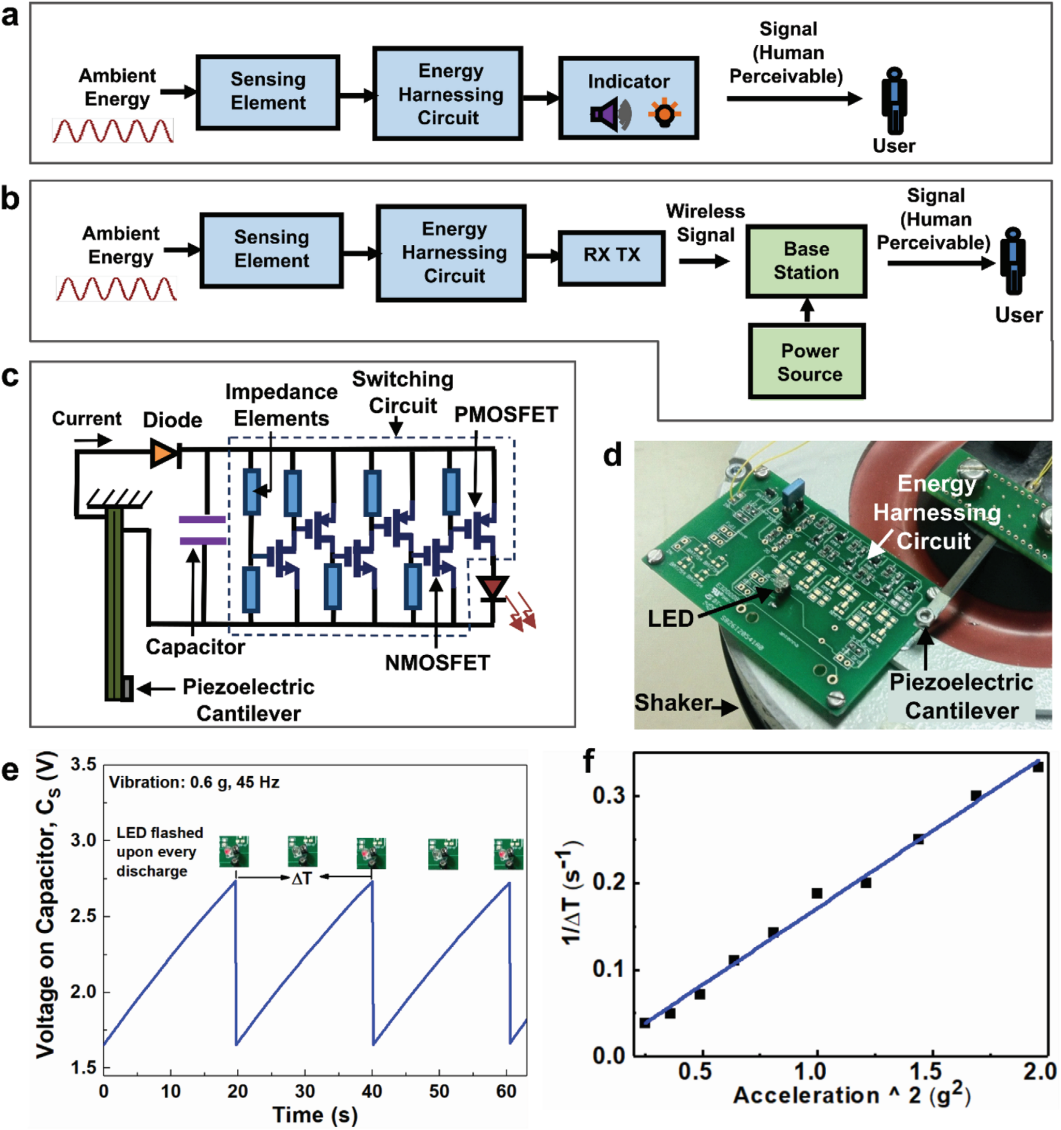
Completely battery‐less and wireless sensor with the entire operation inclusive of result indication driven solely by the sensing element, demonstrated with an accelerometer. a) Conceptual illustration of the completely battery‐less sensor directly generating indicative signal in the form of light or sound directly perceivable by human. b) In contrast, a battery‐less sensor concept based on simultaneous sensing and energy harnessing mechanism generating wireless RF signal instead of directly perceivable signal. c) Circuit implementation of a battery‐less accelerometer producing pulsed light signal indicative of the acceleration magnitude. d) Image of the completely battery‐less accelerometer prototype including LED as the visual indicator. e) Charging and discharging operation of a capacitor driven by piezoelectric cantilever under vibration generating indicative pulsed light at every discharge. f) Frequency (1/Δ*T*) of the pulsed light output by the battery‐less accelerometer exhibits a linear relationship with the square of the acceleration magnitude. Reproduced with permission.^[^ ^197^ ^]^ Copyright 2016, IEEE.

In another example, as illustrated in **Figure** **8**, a battery‐less and wireless sensor concept stipulating the entire self‐powered system operation in a more stringent sense to cover result indication was proposed (as explained in Figure 8a,b). It was implemented with demonstration of a completely self‐powered accelerometer, in which the whole sensor system is driven solely by a ferroelectric sensor. The operation involved harnessing of the mechanical vibration energy into electricity with a piezoelectric cantilever, accumulating the piezoelectric charge, and releasing the charge into an indicator device through an ultra‐sharp switch when the voltage reached a pre‐set threshold value, as implemented with the electrical circuit and prototype exhibited in Figure 8c,d. The vibration condition was quantitatively indicated by the count of pulsed light or sound per unit time (inversely proportional to the pulses’ intervals). At a specified frequency, the square of the acceleration magnitude was proportional to the frequency of the light flash or sound pulses, which is directly perceivable to the user, as illustrated in Figure 8e,f.^[^ ^197^ ^]^

High frequency piezoelectric property at RF range of ferroelectric materials is very useful for realizing wireless system with energy transmitted by electromagnetic wave. As an example, a wireless neural probe system was built for stimulation of neurons and for reading of neural signals in the brain.^[^ ^198^ ^]^ The neural probe system comprised a one‐port ferroelectric LiNbO_3_ SAW reflective delay line, neural firing‐dependent ferroelectric capacitor, two antennas, a static capacitor, Schottky diode, network analyzer and metal tip for stimulating neurons. The probe for reading neural signals employed PVDF ferroelectric capacitor with polarization and volume changed with neural firings. Stimulation amplitude and duration of the resultant output pulses were manipulated by modulating RF input power and the cycle number from interrogator. High sensitivity and linearity were obtained for the output signals in terms of input electrical pulses. Piezoelectric‐based wireless SAW and BAW sensors are an important area in the future IoT applications.^[^ ^199^, ^200^, ^201^, ^202^ ^]^ In piezoelectric SAW and BAW applications, the large electromechanical coupling coefficients of ferroelectric materials offer great value in enhancing the signal noise ratio. Furthermore, many ferroelectric materials possess highly tunable dielectric permittivity up to GHz range, suitable to be used for decoupling and tunable microwave and memory capacitors, as reviewed in the literature and demonstrated with recent progress.^[^ ^203^, ^204^, ^205^, ^206^, ^207^, ^208^ ^]^ Thus ferroelectric materials are promising for playing a significant role in wireless signal transmission for distributed sensor nodes.

### 5.3 Ferroelectrics for Low Power Electronics and Edge Computing

Energy and edge computing are two major techical challenges critical to the success in achieving distributed intelligence, particularly when involving a large number of wireless sensors. For energy issue, a wireless smart system is often required to operate in an environment where energy is scarce or uncertain, and the energy supply may be in intermittent mode. For edge computing issue, the sensors are required to be able to process the data and even make certain decision at local level, rather than send whatever analogue output or the collected data from the sensors to the central system, such as the cloud, for processing and interpretation. The technical approach without any local data processing or analysis ability involves enormous deluge of data transmissions, which is not energy effective and not sustainable for many scalable sensor networks and IoT implementation.^[^ ^209^ ^]^

The multifunctionalities of ferroelectrics provide promising technical solutions to the desired low power electronic circuits and systems, with the ability of utilizing intermittent power supply for edge computing. As ferroelectric sensors can generate electrical output signals by converting the stimuli energy, the most direct method with general applicability for realizing low power wireless operation is to connect and integrate the sensors with a low power CMOS (Complementary Metal Oxide Semiconductor) integrated circuit (IC) for signal processing and data transmission. As proof of concept, a wearable respiration monitoring system by detecting pulsatile vibration was demonstrated, which comprised a ferroelectric PVDF piezoelectric sensor, a reconfigurable analog‐to‐digital converter, a charge amplifier from a standard 130‐nm CMOS process, and an impulse‐radio ultra‐wideband transmitter working in GHz range. The total power consumption of the stable wireless respiration monitoring system was estimated to be roughly 800 µW.^[^ ^210^ ^]^ For the power supply, lithium‐polymer battery of 3.7 V was used, which could be replaced by an energy scavenging device to output power below 1 mW.

Another low power perpetual time recording system to record the time of events was developed for an IoT wireless sensor node use, using FRAM and a real time clock (RTC) powered by a kinetic energy harvester, where a piezoelectric PVDF film was used as the event‐driven sensor. The power‐management circuit remained in sleep mode normally to cut off the power supply to the FRAM as a nonvolatile memory and micro‐processor. However, the RTC alone still required a small standby power of 231 nW from a battery to keep the time for seamless operation. With the PVDF film to generate energy, the power management circuit could supply adequate energy for external circuit to record the event time with required energy of 467 µJ.^[^ ^211^ ^]^

While FRAM provides reliable nonvolatile memory function, with low power consumption compared to most of nonvolatile memories, such as embedded flash memories requiring high voltage for nonvolatile writing, phase change random access memory (PRAM), magneto‐resistive random access memory (MRAM), and resistive random access memory (RRAM),^[^ ^212^, ^213^, ^214^ ^]^ the energy consumption is still higher than static random‐access memory (SRAM) mainly because of its higher access latency. SRAM‐based technical approach, on the other hand, does not provide the desired reliability under the operation with intermittent power supply from energy harvesting. Thus, a hybrid FRAM‐SRAM microcontroller design was proposed and demonstrated to retain the reliability and further obtain lowered energy consumption provided by FRAM and SRAM, respectively, where an energy‐aware memory mapping technique mapped different program sections to the FRAM‐SRAM microcontroller for minimizing energy consumption but not sacrificing reliability. A hardware‐software technique to align the system's powered‐on time intervals to function execution boundaries was adopted for further improving the performance and energy efficiency. The obtained experimental results showed doubled performance with 20% of energy reduction over a FRAM‐based solution.^[^ ^215^ ^]^

Circuit simulations showed that introducing ferroelectric function to SRAM (FE‐SRAM) can significantly mitigate performance degradation to the base SRAM cell to achieve stable dynamic recall operations with only minimal area penalty, and a nonvolatile write energy below 10 fJ bit^−1^ is feasible.^[^ ^216^, ^217^ ^]^ More recently, a new nonvolatile SRAM hybrid model comprises ferroelectric FinFET embedded in a 6‐T SRAM was proposed. The simulation demonstrated superior energy efficiency of a few fJ bit^−1^, low access latency within 1 ns and reduced area penalty.^[^ ^218^ ^]^

To improve efficiency for working with the energy harvested from the environment with intermittent availability, an electrical circuit processor with nonvolatile memory is required to sleep at sudden power loss and to wake up at high speed; otherwise, the overall performance of the nonvolatile processor would degrade significantly in the practical applications. By adopting a hybrid CMOS and backup ferroelectric capacitors, nonvolatile processors based on ferroelectric flip‐flops were demonstrated, which could backup system states within 7 µs and restored signals within 3 µs in the chip level, with a few nanowatt energy consumption during the backup operations. The experimental results demonstrated that the nonvolatile processor could operate continuously under power failure conditions occurring at 20 kHz. The nonvolatile processor had zero standby power and thus highly resilient to power failures.^[^ ^219^ ^]^ For improving the nonvolatile sleep/wake‐up speed at system level, the same team designed a hybrid CMOS/ferroelectric nonvolatile flipflop with a high‐speed voltage detector, integrated in the processor to jointly minimize the overall sleep and wake‐up time. The CMOS/FeRAM nonvolatile system could achieve 46 µs wake‐up time and 14 µs sleep time, with robust nonvolatile operation in the face of power fluctuations.^[^ ^220^ ^]^

These results show that integration of CMOS circuit and FeRAM, including hybrid FE‐SRAM could provide a competitive technical solution for realizing low power distributed sensors, even when they are operated with the intermittent power supply harvested from the uncertain ambient conditions.

**Figure 9:**
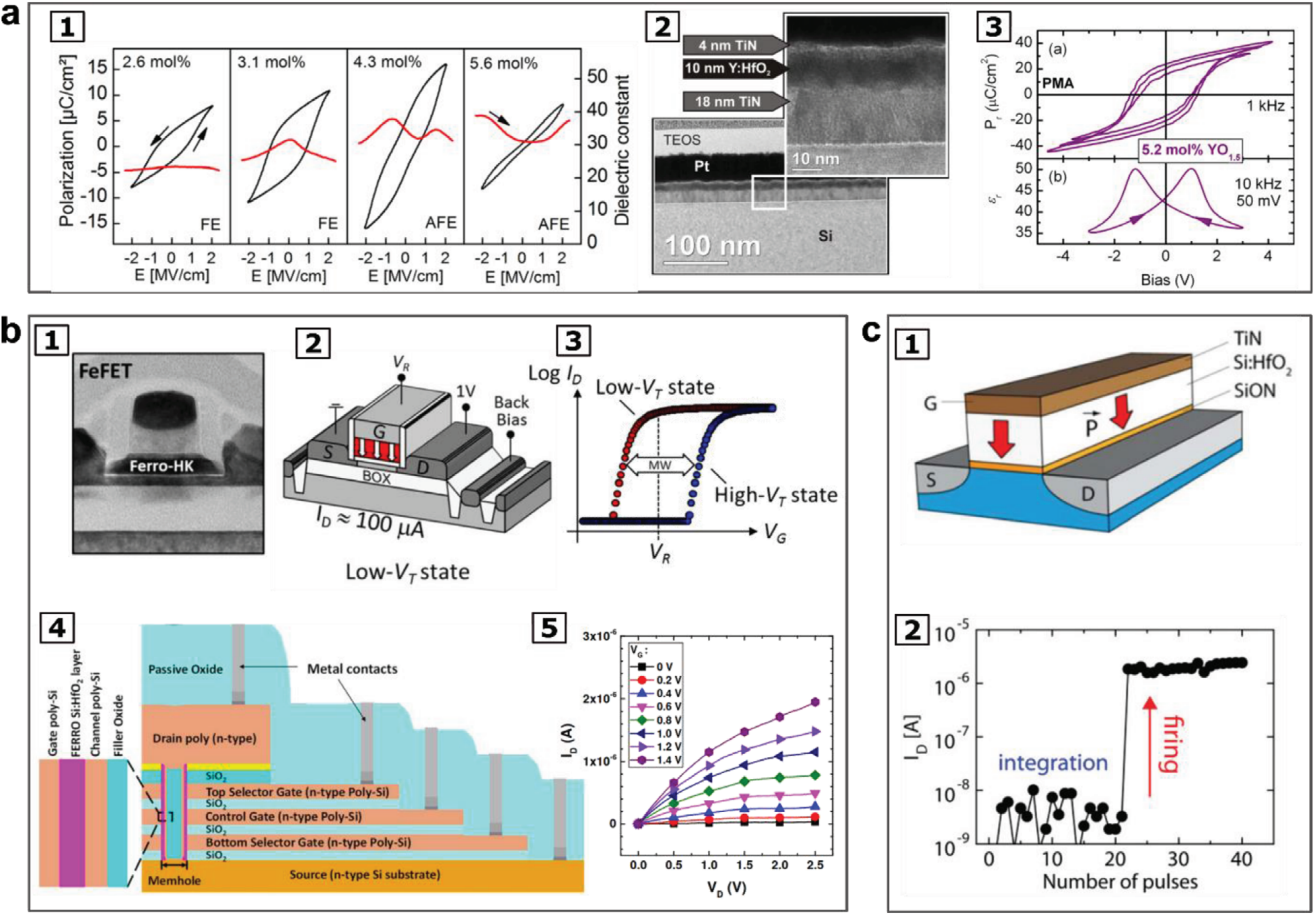
Hafnium oxide‐based ferroelectric field‐effect transistors (FeFET). a) The discovery of ferroelectricity in appropriately doped hafnium oxide (HfO_2_) thin films in 2011: a1) Polarization‐electric field (P‐E) hysteresis loops and curves of dielectric constant‐electric field of Si:HfO_2_ film in different compositions, showing the transition from ferroelectric to anti‐ferroelectric with increasing Si mol%. Reproduced with permission.^[^ ^225^ ^]^ Copyright 2011, AIP Publishing. a2) TEM images showing the cross‐sectional structure of TiN/Y:HfO_2_/TiN stack. a3) P‐E hysteresis loops and curves of dielectric constant‐electric field of Y:HfO_2_ thin film. Reproduced with permission.^[^ ^227^ ^]^ Copyright 2011, AIP Publishing. b) Ferroelectric HfO_2_ film‐based nonvolatile memory: b1) Cross‐section structure of the nonvolatile memory with ferroelectric HfO_2_ thin film integrated in the gate. b2) Diagram of the stable polarization states of the ferroelectric layer in the memory device. b3) Drain‐current/gate‐voltage (*I*_D_–*V*_G_) curve of the device, wherein the two stable polarization states are observed. Reproduced with permission.^[^ ^154^ ^]^ Copyright 2017, IEEE. b4) Diagram of the cross‐section structure of the macaroni‐type 3‐D FeFET‐based nonvolatile memory. b5) Output characteristics (*I*_D_–*V*_D_) of the 3‐D FeFET‐based nonvolatile memory, exhibiting typical behaviour of scaled MOSFET devices. Reproduced with permission.^[^ ^155^ ^]^ Copyright 2018, IEEE. c) Nanoscale ferroelectric HfO_2_‐based FeFETs emulating neuronal behaviours for multistate neuromorphic computing application: c1) Schematic illustration of the HfO_2_‐based FeFET structure. c2) The transition from Off state to On state in the form of accumulative switching induced by continuous input stimuli. Reproduced with permission.^[^ ^228^ ^]^ Copyright 2018, RSC Publishing.

As described in Section 4.2, the combination of the three terminal transistor configuration and bi‐stable spontaneous polarization states makes it feasible to achieve further reduced power consumption on FeFET at the scale of 1 fJ bit^−1^. Many ferroelectric thin films have been applied to produce FeFET since 1990s.^[^ ^221^, ^222^, ^223^ ^]^ However, the ferroelectric thin films used for FeFEM often suffered incompatibility issues with the current silicon‐based high density IC technology, such as high processing temperature, degradation of ferroelectricity with size reduction,^[^ ^224^ ^]^ and thus the scale of FeFET application is restricted. In 2011, it was found that appropriately doped hafnium oxide (HfO_2_) thin films are ferroelectric, and are ideally suited for FeFET due to its excellent compatibility to CMOS,^[^ ^225^ ^]^ although bulk HfO_2_ is not a ferroelectric material. The HfO_2_‐based ferroelectric thin films can form conformal ultrathin layer structure, maintain ferroelectricity down to a few nanometers, and processed at a temperature below 450°C.^[^ ^226^, ^227^ ^]^ **Figure** **9** presented the HfO_2_ thin films, including the polarization‐electric field hysteresis loops and dielectric constant‐electric field curves of HfO_2_ thin films of different compositions as exhibited in Figure 9a. The application of HfO_2_ thin film in FeFET for achieving two stable polarization states is illustrated in Figure 9b, demonstrating its viability as a nonvolatile memory device. Nanoscale ferroelectric HfO_2_‐based FeFETs can even emulate neuronal behaviors for multistate neuromorphic computing applications, as illustrated in Figure 9c, such that accumulative switching can be realized under continuous input stimuli.^[^ ^228^ ^]^

After analyzing the potential of the FeFET technologies in embedded nonvolatile memory applications, and future in‐memory, biomimetic and various computing models, recent technical reviews show that HfO_2_‐based FeFET and its enabled ferroelectronics are promising key hardware components for the future ultralow power edge computing.^[^ ^209^, ^229^ ^]^ The progress in achieving stable ferroelectricity in ultrathin oxides with simple composition and low processing temperature compatible with CMOS manufacturing processes is exhibiting a bright future in realizing ferroelectric logic and memory functionalities at the single‐device level. This is promising for offering competitive ultralow power in‐memory computing capabilities for implementing the algorithm and realizing distributed AI function for IoT system.^[^ ^209^ ^]^

**Figure 10:**
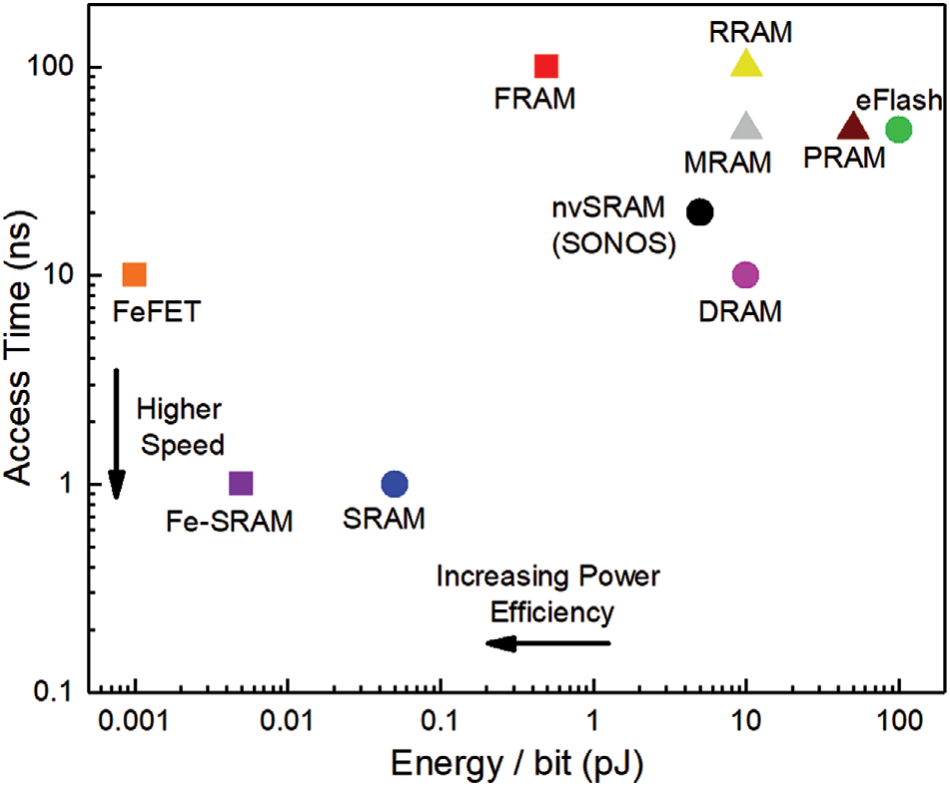
Access speed and energy profiles of ferroelectric‐based, mainstream embedded and other emerging memory devices.

The energy consumption and access speed of various memory technologies are mapped out in **Figure** **10**. The comparisons on other metrics, including endurance, nonvolatile and nondestructive read characteristics are summarized in **Table** **3**. Currently, FeFET under development is leading in the energy reduction as compared to other nonvolatile memory technologies and possesses superior access speed approaching that of the commercially available volatile SRAM. The attractive energy and speed profile of FeFET, in conjunction with its nondestructive read and viable endurance characteristics, promises a key enabling technology towards highly energy efficiency and very low latency nonvolatile memory device for edge computing under low and intermittent power.

**Table 3:** Comparison of energy, endurance, nonvolatile and nondestructive read characteristics of ferroelectric‐based memories with other technologies. Devices’ characteristics are obtained from references.^[^ ^209^, ^212^, ^214^, ^218^, ^229^ ^]^

	Memory Devices	Nonvolatile	Energy/bit (pJ)	Endurance^a)^	Nondestructive Read
Ferroelectric Based	FRAM	Yes	0.1–1	M	No
	FeFET^b),c)^	Yes	0.001	L to M	Yes
	FE‐SRAM^b),c)^	Yes	0.001–0.01	L to M	Yes
Mainstream Embedded Memory	eFlash	Yes	100	L	Yes
	SRAM	No	0.01–0.1	H	Yes
	nvSRAM (SONOS)	Yes	1–10	L	Yes
	DRAM	No	10	H	Yes
Other Emerging Memory	PRAM	Yes	10–100	L	Yes
	MRAM	Yes	10	L	Yes
	RRAM	Yes	10	L	Yes

^a)^ H: > 10^15^ cycles; M: 10^15^–10^9^ cycles; L: < 10^9^ cycles

^b),c)^ Under research.

For the various existing nonvolatile memories, backup and restore operation takes a substantial amount of time and energy caused by delay and dissipation due to the existence of the capacitance and resistance in the circuitry.^[^ ^230^, ^231^ ^]^ By taking use of the so‐called negative capacitance effect of a ferroelectric thin film, low voltage nonvolatile memory comprising NCFET potentially with orders of magnitude energy reduction for backup and restore operation has been proposed. The NCFET device operates in a cross‐coupled circuitry that can avoid static drain–source current during backup and restore operation, while a further miniaturization can maintain fast and robust operations with a sharp and stable On/Off states. Theoretical simulation results showed that, for the memory comprising negative capacitor made of PZT on well matched HfO_2_ buffer layer with scale at 10 nm, the time for the backup and restore operation can be reduced to well below 1 nsec, with energy consumption on the order of 1 fJ.^[^ ^232^ ^]^

**Figure 11:**
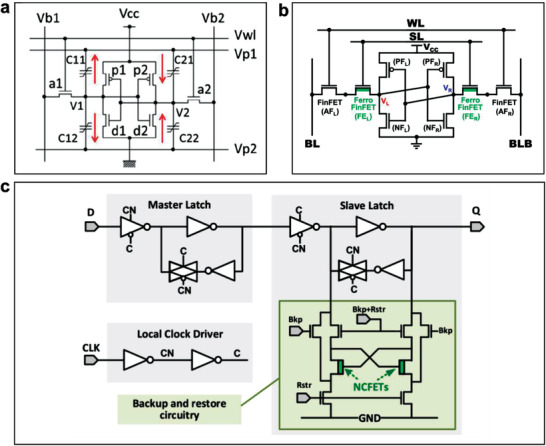
Ferroelectric hybrid memory device for nonvolatile computing under low and intermittent power: a) FRAM‐SRAM consisting of 4 FRAMs in a 6‐transistor SRAM whereby data registered in SRAM is backed up to FRAMs by the store operation before power cutoff, and is transferred back to SRAM by the recall operation when power resumed. Reproduced with permission.^[^ ^217^ ^]^ Copyright 2019, IEEE. b) FeFET‐SRAM consisting of 2 Fe‐FET in a 6‐transistor SRAM, which utilizes ultra‐low power and fast switching FeFETs as the nonvolatile backup storage to minimize energy consumption and latency delay during the store and recall cycles. Reproduced from reference^[^ ^218^ ^]^ under the terms of the CC‐BY Creative Commons Attribution 4.0 International License (https://creativecommons.org/licenses/by/4.0) (CC BY 4.0). c) NCFET‐DFF memory device consisting of a standard D flipflop and a pair cross‐coupled nonvolatile NCFETs, whereby the NCFET possesses steep‐switching capability arising from negative capacitance effect and low damping coefficient of the ferroelectric gate materials, to significantly reduce the energy and latency of the backup memory function. Reproduced with permission.^[^ ^232^ ^]^ Copyright 2017, IEEE.

**Figure** **11** presented the ferroelectric hybrid memory device designs, including FRAM‐SRAM (Figure 11a), FeFET‐SRAM (Figure 11b), and NCFET‐ D‐flipflop (DFF) (Figure 11c), as mentioned above. By introducing ferroelectric materials into conventional CMOS memory devices, data backup operation under intermittent power can be enabled by the ferroelectric nonvolatile stable states. During power availability, the data registered by the CMOS memory portion is copied into the nonvolatile ferroelectric memory by the store operation. Upon power outage, the data is retained in the ferroelectric memory held by one of the two nonvolatile stable states. As power resumes, the data is then copied back from the ferroelectric memory to CMOS memory portion to enable the continuity in accumulative computation.

However, the speed and energy consumption performance of memory devices depend on the physical parameter of real ferroelectric materials that can be obtained. The damping constant of ferroelectric thin film is the determinant of operation frequency of FeFET for utilizing the potential low power advantage from the design. Only by using the ferroelectric thin film with an adequately low damping constant and shorter delay than intrinsic MOSFET, FeFET can further significantly reduce device energy consumption beyond the state‐of‐the‐art CMOS technology.^,[^ ^233^ ^][^ ^234^ ^]^

As neuromorphic computing shows many advantages in performing data‐centric tasks in terms of energy efficiency and learning ability, studies are motivated to develop artificial synapses that are able to effectively emulate the corresponding multiple functionalities exhibited by biological counterparts.^[^ ^235^, ^236^, ^237^, ^238^, ^239^ ^]^ Ferroelectrics have exhibited great potential for neuromorphic computing. Z. Wang et al experimentally demonstrated FeFET based spiking neurons, with dual neuron functionality of both excitatory and inhibitory input connections, as a key feature required for bio‐mimetic neural networks for unsupervised learning. A spiking neural network for unsupervised clustering was implemented, benchmarking the network performance across analog CMOS and emerging technologies, showing a classification accuracy of 93%, and low power of 3.6 nW during classification.^[^ ^240^ ^]^ With a compact dynamical model of a FeFET based spiking neuron that can capture the dynamic behavior of the FeFET neuron including the spike timing and frequency, Y. Fang et al showed that the FeFET based spiking neuron, with different excitatory and inhibitory inputs, could imitate various spiking patterns for cortical neurons.^[^ ^241^ ^]^ L. Chen et al demonstrated three‐terminal synaptic transistor made of an inorganic ferroelectric Hf_1−x_Zr_x_O_2_ gate stack integrated with a 2D layered semiconductor (WS_2_) for emulating the synaptic plasticity. Besides a large memory window and a current on/off ratio of about 10^5^ between the two states, the device exhibited bio‐mimetic feature of synaptic plasticity, such as potentiation, spike amplitude dependent plasticity (SADP), depression, and spike‐rate dependent plasticity (SRDP).^[^ ^242^ ^]^ An analog synaptic device was fabricated using nanoscale ferroelectric HfZrO_x_/TiN and indium gallium zinc oxide (IGZO) FeFET structure (called ferroelectric transistor in the original work), where the conductance of the semiconductor channel was controlled by ferroelectric polarization. The potentiation and depression properties in FeFET were evaluated with incremental bias pulses. An artificial neural network simulation using the measured results exhibited 91.1% recognition accuracy in recognizing handwritten digits, exhibiting the potential for developing the neuromorphic hardware systems based on FeFET.^[^ ^243^ ^]^

## 6 Opportunities and Challenges for Ferroelectric Materials in Distributed Intelligence

According to estimation by Fortune Business Insights, the giant global IoT market size is rapidly growing at a compound annual growth rate (CAGR) of 24.9%, from US$ 250.72 billion in 2019 to US$ 1463.19 billion in 2027.^[^ ^244^ ^]^ The increasing adoption of digital twins, which are transforming the physical assets in a wide range of industries into virtual representations for monitoring and controlling the physical assets through the digital platform, is providing a significant impetus for the market growth. Minimizing physical presence could even mitigate the impact of outbreak of the COVID‐19 pandemic and minimize the losses due to social distancing constraint and lockdown by the relevant rules as implemented. Even not always connected to the internet, other smaller market sectors related to nondestructive and continuous sensing and monitoring technologies are also growing rapidly. For example, demand for structural health monitoring (SHM) is increasing as the technology can realize structural state awareness and thus facilitate appropriate preventative repair and maintenance schemes in reference to data from the distributed sensors during the service life. Applied Market Research estimated that the global SHM market size has a CAGR of 14.5% from 2020 to 2027, from US$1.674 billion in 2019 to reach US$3.815 billion in 2027, even with the impact of COVID‐19.^[^ ^245^ ^]^ While wired sensors will continuously be widely used in the market, wireless SHM technology is expected to grow at the highest CAGR during the forecast period, with their many advantages in terms of easier sensor installation and data transferring by eliminating the cables.^[^ ^245^ ^]^

Ultralow power and even self‐powered wireless sensors that are characterized with pulsed signal transmission mode, ability to harvest and operate with intermittent scarce energy in the environment, and local computing capacity, are particularly desired for realizing the distributed intelligence as demanded in digitalized smart systems and IoT. This provides ferroelectric materials the tremendous market opportunity, considering their in‐principle zero‐energy sensing and multiple energy harvesting mechanisms, direct electrical energy storage with high power pulsed‐mode output ability, and ultralow power nonvolatile memory function. In the meantime, advancements in signal processing algorithm and artificial intelligence will be able to improve the interpretation on the signals of ferroelectric sensors and better connect the state or condition under monitoring with the sensors’ outputs.

While the tremendous application demand brings about great opportunities, material scientists must overcome the material‐centric technical challenges in order to effectively expand the applications of ferroelectric material in distributed intelligence and IoT.

Firstly, ferroelectric materials with the compositions, scalability and processing method compatible with the implementation environment and conditions are to be developed for realizing widely distributed energy autonomous sensing and energy harvesting devices. With the expanding applications and increasing concerns on lead hazard, the pressure from possible legislation restriction of lead in ferroelectric and piezoelectric devices are driving industry to seriously seek for lead‐free material alternatives.^[^ ^246^, ^247^ ^]^ Replacing existing lead‐based ferroelectric materials with lead‐free compositions is an urgent task for obtaining immediate solution as preferred. To facilitate distributed sensor and energy harvesting transducer implementation, scalable ferroelectric thin films and coatings are desired for microelectromechanical system (MEMS) sensors, flexible sensors, and in‐situ fabricated sensors. MEMS sensors are produced through scalable wafer‐based batch microfabrication process to realize high level device miniaturization at lowered unit cost.^[^ ^248^ ^]^ By different approaches for combining MEMS sensors with ICs, including the hybrid multiple chip and system‐on‐chip solutions through monolithic integration and heterogeneous integration techniques, MEMS sensors can be packaged and integrated with ICs with system functionalities including the signal processing, edge computing, and wireless communication. In the recent decade, the use of piezoelectrics in MEMS or piezo‐MEMS is accelerating due to the advantages of material‐inherent electromechanical response, mainly enabled with perovskite ferroelectric and piezoelectric thin films investigated since 1990s.^[^ ^249^, ^250^, ^251^, ^252^, ^253^ ^]^ Flexible sensors and transducers offer the structural flexibility, conformability, wearability and compatibility with organic electronics, and thus have obtained great attention for wearable device applications.^[^ ^254^ ^]^ The flexibility and large structural area lead to better responsivity to low frequency mechanical inputs and are often more effective for energy harvesting from environment.^[^ ^255^, ^256^, ^257^ ^]^ For improving reliability and reducing cost by eliminating the tedious work and human error factors involved during manual installation process, sensors and transducers produced through in‐situ fabrication or direct write process are emerging.^[^ ^258^, ^259^, ^260^, ^261^, ^262^ ^]^ When stable mechanical or acoustic coupling between the sensors and the structure to be monitored is essential, such as in acoustic emission monitoring and active ultrasonic monitoring, implementation of piezoelectric transducers by in‐situ fabrication becomes particularly attractive. The reason is that this approach provides a reliable way to determine the change of acoustic signatures versus service time or event occurrence. This is an area where lead‐free ferroelectric ceramics can make great contributions,^[^ ^263^, ^264^, ^265^ ^]^ as shown with the development of scalable processing of lead‐free piezolectric coatings such as by thermal spray process. It is almost impossible to accept the toxic lead compositions in the high temperature processing in open air over large area during the thermal spray process.^[^ ^247^, ^266^, ^267^, ^268^ ^]^

Secondly, ferroelectric materials with superior performance are to be obtained, including adequate response magnitude for directly driving energy autonomous sensor system operation and retaining the significant ferroelectric effect at low dimensions for high density computing function integration. While ferroelectrics possess the in‐principle zero‐energy sensing mechanism by directly converting the stimuli into electricity, the electrical output is rather low (in reference to the data as shown in Tables 1 and 2). The small primary sensor output may not be an issue for the operation of conventional sensors with external circuits including amplifiers and power supplies present. However, such small primary electrical outputs are often not large enough to drive a typical sensor system without external power supply. In the recent years, ferroelectric materials with significantly improved performance properties are produced, promising for significantly upgrading device responsive output. For bulk ferroelectric materials, giant piezoelectric coefficient *d_33_* values ranging from 3400 to 4100 pCN^−1^ have been obtained in relaxor ferroelectric single crystals by enhancing local structural heterogeneity through rare‐earth doping.^[^ ^269^ ^]^ Organic‐inorganic hybrid molecular crystals with large electric‐field driven strain have been synthesized.^[^ ^270^, ^271^ ^]^ It is well known that many ferroelectric bulk materials with perovskite structure may suffer degradation and loss of their ferroelectric properties at reduced sizes at the nanoscales. However, there are exciting new opportunities at the small scales, as observed in the recent years. It is evident that some simple compositions that are conventionally not ferroelectric in the bulk become ferroelectric at such a small size that has previously been thought impossible for the existence of ferroelectricity,^[^ ^272^ ^]^ particularly including the HfO_2_‐based ferroelectrics.^[^ ^273^, ^274^ ^]^ One study on low dimensional HfO_2_ shows that flat energy bands exist and induce robust and switchable dipoles. The intrinsically stable, switchable and localized electric dipoles means distinct ferroelectricity within half‐unit cell widths down to angstrom scale, which exhibits the potential for realizing unit cell‐by‐unit cell ferroelectric switching or memory devices integrated with the ultimate high density silicon‐based IC.^[^ ^275^, ^276^ ^]^ It is found that conventional anti‐ferroelectric sodium niobate in bulk can become ferroelectric thin film due to self‐assembled nano‐column structure and loss of local chemical stoichiometry, which exhibits ferroelectricity with giant electromechanical response beyond the piezoelectric response of its bulk counterpart.^[^ ^277^ ^]^ With high‐energy ion bombardment to generate intrinsic point defects, and thus to improve breakdown strength and manipulate polarization at different electric field, energy storage densities as high as ∼133 J cm^−3^ has been observed in relaxor ferroelectric thin films with nano‐scaled domains.^[^ ^144^ ^]^ These extraordinary properties of new or structure‐modified ferroelectric materials could lead to exciting sensors, transducers and ferroelectronic systems in the future with significantly upgraded performance. For realizing the great potential of NCFET for further reducing energy consumption, it is critical for material scientists to discover and produced ferroelectric thin films with significantly lowered damping effect.

Thirdly, the gap between material performance property and device system viability is to be bridged with multidisciplinary knowledge and design. The end users of various smart sensor systems and IoT make their choices of technologies for adoption based on the performance and cost at system level. While the multifunctionalities of ferroelectrics have the great potential to enable radical innovations on smart systems and IoT technologies, there is still the gap between the potentials of the multifunctionalities offered by ferroelectrics and the competitiveness of smart systems for commercial deployment. Multidisciplinary knowledge and competencies, from material science, electronic engineering, data processing, system design, artificial intelligence and even metrology standards are required to achieve the smart systems and IoT technology enabled by ferroelectric multifunctionalities. Collaborations with the multidisciplinary knowledge and competencies are highly demanded for realizing the material‐critical breakthrough with technical competitiveness and commercial viability at the system level.

## 7 Conclusion

Ferroelectric materials possess extraordinary multiple signal or energy conversion and storage functions. They have achieved many applications enabled by various individual functions, such as electromechanical sensors and transducers enabled by piezoelectric effect, thermal detectors and imaging sensors by pyroelectric effect, optical sensors by bulk photovoltaic effect, capacitors and nonvolatile memories by the high dielectric permittivity and switchable spontaneous polarization. As sensors, ferroelectric materials can generate the signal output by directly converting the energy of various stimuli to be detected without requiring external power supply in principle. As energy transducers, ferroelectric materials can harvest multiple forms of energy with high reliability and durability with their thermodynamic stable polar phases. As capacitors, ferroelectric materials exhibit the great values in realizing high power density electrical storage with high charging/discharging rate and ability of pulse‐mode signal generation. The nonvolatile memories derived from ferroelectrics are able to realize digital processors and systems with ultralow power consumption, sustainable operation with intermittent power supply, and neuromorphic computing. These characteristics are invaluable for realizing distributed sensors and smart systems.

Many of the examples as cited and analyzed here have further showed that the multiple functionalities of ferroelectric materials can be combined with synergy to achieve wireless, ultralow‐power or even self‐powered sensors and operation systems, which are particularly demanded for realizing distributed intelligence in cyber‐physical interactive applications including IoT technology. It is highlighted that radical device innovations with the ferroelectric‐enabled technologies are often largely promoted by the discovery of new ferroelectric materials, or conceptual breakthrough based on insightful design with synergistic connections among the multifunctionalities of ferroelectric materials. Discoveries of new compositions and structures to obtain ferroelectric materials with dramatically improved performance, such as at low dimensions, will continuously be expected for the future. Multidisciplinary expertise and interdisciplinary collaborations, not only involving deep understanding on the underlying material properties and multifunctionalities of ferroelectrics, but also on the latest technical advancement in other areas, including microelectronics, signal processing, artificial intelligence, and metrology standard, are required for realizing the material‐critical breakthrough with technical competitiveness and commercial viability at the system level.

## Conflict of Interest

The authors declare no conflict of interest.
